# Quinazolin-4-one/3-cyanopyridin-2-one Hybrids as Dual Inhibitors of EGFR and BRAF^V600E^: Design, Synthesis, and Antiproliferative Activity

**DOI:** 10.3390/ph16111522

**Published:** 2023-10-26

**Authors:** Lamya H. Al-Wahaibi, Mohamed Hisham, Hesham A. Abou-Zied, Heba A. Hassan, Bahaa G. M. Youssif, Stefan Bräse, Alaa M. Hayallah, Mohamed Abdel-Aziz

**Affiliations:** 1 Department of Chemistry, College of Sciences, Princess Nourah Bint Abdulrahman University, Riyadh 11564, Saudi Arabia; lhalwahaibi@pnu.edu.sa; 2 Pharmaceutical Chemistry Department, Faculty of Pharmacy, Deraya University, Universities Zone, New Minia City 61111, Egypt; mohammedhisham90@yahoo.com (M.H.); drhesham92@yahoo.com (H.A.A.-Z.); 3 Medicinal Chemistry Department, Faculty of Pharmacy, Minia University, Minia 61519, Egypt; heba.hasan@mu.edu.eg (H.A.H.); abulnil@hotmail.com (M.A.-A.); 4 Pharmaceutical Organic Chemistry Department, Faculty of Pharmacy, Assiut University, Assiut 71526, Egypt; 5 Institute of Biological and Chemical Systems, IBCS-FMS, Karlsruhe Institute of Technology, 76131 Karlsruhe, Germany; 6 Pharmaceutical Organic Chemistry Department, Faculty of Pharmacy, Sphinx University, Assiut 71515, Egypt

**Keywords:** quinazolin-4-one, 3-cyanopyridin-2-one, EGFR, BRAF^V600E^, anti-cancer, docking

## Abstract

A novel series of hybrid compounds comprising quinazolin-4-one and 3-cyanopyridin-2-one structures has been developed, with dual inhibitory actions on both EGFR and BRAF^V600E^. These hybrid compounds were tested in vitro against four different cancer cell lines. Compounds **8**, **9**, **18**, and **19** inhibited cell proliferation significantly in the four cancer cells, with GI_50_ values ranging from 1.20 to 1.80 µM when compared to Doxorubicin (GI_50_ = 1.10 µM). Within this group of hybrids, compounds 18 and 19 exhibited substantial inhibition of EGFR and BRAF^V600E^. Molecular docking investigations provided confirmation that compounds **18** and **19** possess the capability to inhibit EGFR and BRAF^V600E^. Moreover, computational ADMET prediction indicated that most of the newly synthesized hybrids have low toxicity and minimal side effects.

## 1. Introduction

Cancer is still a heavily researched public health issue in modern civilization. Despite intensive research, it remains a significant cause of death in wealthy countries [1,2,3]. Cancer’s danger rests in its capacity to infiltrate and destroy normal tissues and organs, causing them to malfunction. Furthermore, cancer can spread to other body regions, complicating treatment [4,5,6]. Cytotoxic therapy has long been considered the gold standard in cancer treatment [7]. However, due to inconsistencies in treatment outcomes and inadequate safety, new approaches to cancer treatment have evolved [8,9,10]. The development and widespread clinical testing of precisely targeted anti-cancer tools such as therapeutic antibodies [11], tyrosine kinase inhibitors (TKIs) [12,13,14], micro-RNA therapy [15], oncolytic viruses [16], and gene-editing treatments [17] have significantly expanded the arsenal of weapons available to combat various tumor types.

Central to these advancements is the Epidermal Growth Factor Receptor (EGFR), also known as HER1, a transmembrane receptor belonging to the ErbB family alongside HER2, HER3, and HER4 [18,19]. EGFR, a pivotal tyrosine kinase receptor, plays indispensable roles in various physiological processes, including cell cycle regulation [20], differentiation [21], and reorganization of the cytoskeleton [22]. It is frequently overexpressed in several cancer types, where it participates in activities such as cell proliferation, migration, invasion, and angiogenesis [23,24]. Mutations in EGFR are frequent in diseases such as non-small cell lung cancer, head and neck cancer, and colorectal cancer [25,26,27].

Conversely, the BRAF gene encodes BRAF kinase, a cytosolic serine/threonine kinase crucial in cell signaling, growth, and survival pathways [28,29]. BRAF mutations are commonly seen in melanoma, thyroid cancer, and colorectal cancer [30,31].

**Figure 1:**
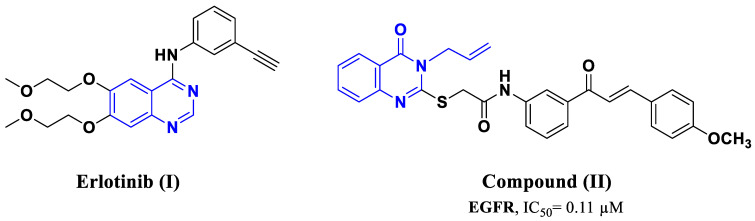
Structure of compounds **I** and **II**.

Nitrogen-based heterocycle molecules are a useful source of necessary building blocks for developing novel bioactive chemicals. *N*-Heterocyclic components are found in over three-quarters of the medications approved by the Food and Drug Administration (FDA) [32]. Quinazoline and quinazolin-4-one are crucial nitrogen-based heterocycles that have been extensively researched in numerous research activities revealing their adaptive biological effects [33,34]. These chemically basic compounds possess a wide range of medicinal properties, such as anti-cancer [33,35], anti-tubercular [36], anti-inflammatory [37], and antimicrobial activities [38]. Many quinazoline-based anti-cancer agents were granted FDA approval and are in clinical use for cancer management, such as Erlotinib (**I**) (Figure 1), demonstrating remarkable potency in inhibiting growth factor receptor tyrosine kinases, particularly targeting the EGFR receptor, and featuring quinazoline scaffolds [39]. Its anti-cancer effectiveness stems from its ability to hinder intracellular phosphorylation of tyrosine kinases at the ATP binding site of the receptor, block JAK2V617F—a mutant variant of JAK2, and initiate pathways that lead to apoptotic cell death [40]. Despite its notable efficacy, specificity, and favorable safety record, patients frequently acquire resistance to this treatment within 8–12 months of initiating therapy due to mutations occurring in the ATP binding site of the EGFR kinase domain [41]. In our recent publication, compound (**II**) with a quinazolin-4-one nucleus had robust antitumor activity against four cancer cell lines, as well as significant dual EGFR and BRAF^V600E^ inhibitory action (IC_50_ = 0.11 µM and 0.65 µM, respectively) (Figure 1) [33].

**Figure 2:**
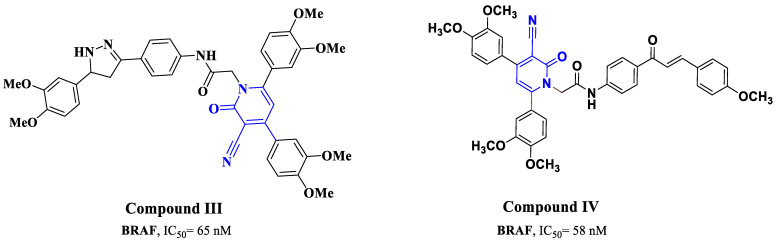
Structure of compounds **III** and **IV**.

Cyanopyridine derivatives have exhibited favorable characteristics in terms of antimicrobial [42], antioxidant [43], antibiotic [44], anti-inflammatory [45], anticonvulsant [46], as well as anti-cancer potential [47,48]. Because of their potential to interact with a variety of biological targets, including tubulin [49], the survivin protein [50,51], and PIM-1 Kinase [52,53], these compounds have received much interest for their anti-cancer characteristics. In our recent publication, 3-cyanopyridone/pyrazoline hybrid **III** (Figure 2) exhibited impressive dual inhibitory efficacy against EGFR and BRAF ^V600E^ (IC_50_ = 68, 65 nM, respectively), with remarkable GI_50_ values of 25 nM. Like Erlotinib, compound **III** displayed a potent inhibitor especially against both cancer cell proliferation and BRAF^V600E^ [54]. In another study, compound **IV** (Figure 2) demonstrated significant efficacy in inhibiting cancer cell proliferation (with a GI_50_ value of 0.72 µM) and promising inhibitory potential against BRAF^V600E^ (with an IC_50_ value of 58 nM), outperforming Erlotinib (with an IC_50_ value of 65 nM) [47].

A challenging issue that has arisen during EGFR inhibitor therapy is the emergence of the BRAF^V600E^ mutation as a potential resistance mechanism [55]. Inhibiting BRAF can also activate EGFR, reviving tumor growth [56,57,58]. Consequently, a strategic approach involving the dual inhibition of EGFR and BRAF^V600E^ has been employed to circumvent these complexities. Several clinical trials, including patients with metastatic colorectal cancer with the BRAF^V600E^ mutation, revealed that the combination of BRAF^V600E^ and EGFR inhibitors was clinically successful [55,59,60].

**Figure 3:**
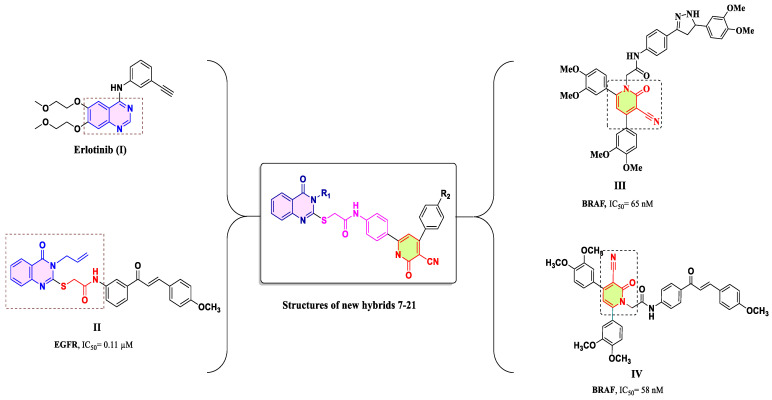
Rational design of newly designed hybrids **7**–**21**.

In our ongoing research to develop antiproliferative hybrids inspired by the biological properties of compounds **I**–**IV**, we hypothesized that these hybrids might possess dual EGFR/BRAF^V600E^ pharmacophoric elements. As shown in Figure 3, the quinazolin-4-one moiety (as seen in compounds **I** and **II**), spacer, and 3-cyanopyridin-2-one moiety (as seen in compounds **III** and **IV**) were identified as important components. As a result, combining these pharmacophores into a single compact structure can lead to the development of potent antiproliferative drugs with EGFR/BRAF^V600E^ inhibitory actions. This approach may provide benefits such as reduced drug resistance development and fewer associated side effects.

As a result, a novel series of quinazolin-4-one/3-cyanopyridin-2-one hybrids (**7**–**21**, Figure 3) were developed and synthesized as dual EGFR/BRAF^V600E^ inhibitors with antiproliferative activity. The newly synthesized compounds were evaluated against a panel of four human cancer cell lines. The most potent compounds were then investigated as EGFR and BRAF^V600E^ inhibitors. Finally, molecular docking studies were carried out to evaluate their binding mechanisms and interactions inside the active sites of molecular targets.

## 2. Results and Discussion

### 2.1. Chemistry

**Scheme 1:**
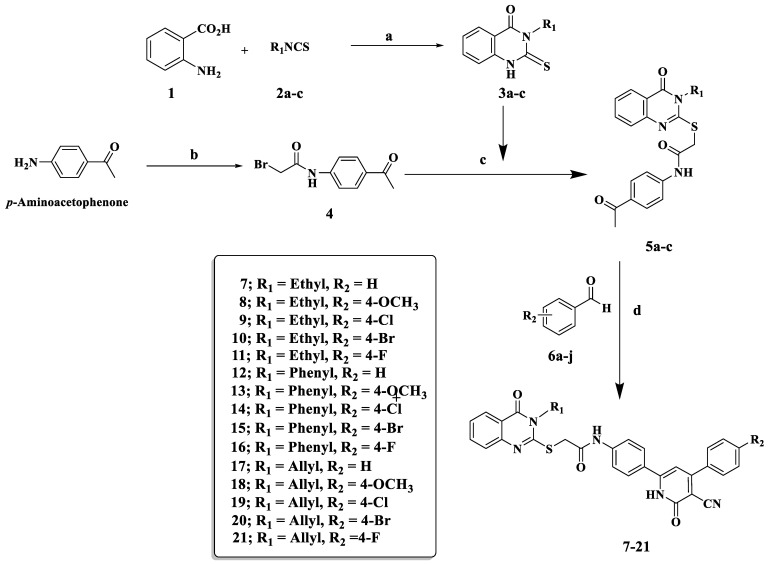
Synthesis of quinazolin-4-one/3-cyano-2-pyridone hybrids (**7**–**21**); reagents and reaction condition: (**a**) TEA/EtOH reflux 1.5 h; (**b**) BrCOCH_2_Br, CH_2_Cl_2_, K_2_CO_3_, H_2_O; (**c**) CH_3_CN, TEA reflux 8 h; (**d**) ethyl cyanoacetate, ammonium acetate, fusion 120–130° for 1.5 h.

The reaction sequences shown in Scheme 1 were used to prepare compounds **7**–**21**. Anthranilic acid **1** was reacted with appropriate ethyl/phenyl/allyl isothiocyanates (**2a**–**c**) in refluxing ethanol in the presence of triethylamine (TEA) as a base catalyst to generate 2-mercapto-3-substituted-(3*H*)-quinazolin-4-ones (**3a**–**c**) [33,35]. *N*-(4-Acetylphenyl)-2-bromoacetamide **4** was synthesized in high yield according to the previously described procedure [61] by treating *p*-aminoacetophenone with bromoacetyl bromide in water and methylene chloride bilayer solvent in the presence of potassium carbonate. The structure of product **4** was confirmed by the reported melting point [61]. Consequently, refluxing compounds **3a**–**c** with *N*-(4-acetylphenyl)-2-bromoacetamide **4** in acetonitrile in the presence of TEA for 8–12 h, and the corresponding intermediates **5a**–**c** were prepared in 75–85% yield. The melting points of compounds **5a**–**c** were confirmed by a previously reported study [35].

The synthesis of quinazolin-4-one/3-cyano-2-pyridone hybrids **7**–**21** was carried out by a one-pot, four-component reaction of equal amounts of acylated quinazolin-4(3*H*)-one **5a**–**c**, ethyl cyanoacetate, suitably substituted benzaldehyde, and an excess of ammonium acetate in absolute ethanol with vigorous stirring at 120–130 °C for 1.5 h to obtain final conjugates **7**–**21**. Regarding the ^1^HNMR of compound **10**, it exhibited a singlet signal at δ: 4.26 ppm, illustrating the presence of methylene group of SCH_2_ protons as well as the presence of a singlet signal at δ: 6.77 ppm corresponding to cyanopyridine H-5. Furthermore, the N**H** group of the 2-pyridone ring appeared as a singlet at δ: 12.75 ppm. In the ^13^C NMR of compound **10**, a signal appeared at δ: 115.26 ppm corresponding to the nitrile group, and a signal at δ: 160.21 ppm related to the carbonyl group of the 2-pyridone ring.

### 2.2. Biology

#### 2.2.1. In Vitro Anti-Cancer Activity

##### Cytotoxicity Assay in Non-Tumorigenic MCF-10A Cells

To evaluate the potential cytotoxic effects of the synthesized compounds on non-cancerous cells, we utilized the human mammary gland epithelial cell line, MCF-10A [62]. Compounds **7**–**21** were incubated with MCF-10A cells for a period of 4 days. After this incubation period, the cell survival was assessed through the 3-(4,5-dimethylthiazol-2-yl)-2,5-diphenyltetrazolium bromide (MTT) assay [9]. Importantly, at a concentration of 50 µM, the investigated compounds did not manifest any cytotoxic effects on MCF-10A cells. For most of the compounds tested, the cell viability remained high, surpassing 86% Table 1.

##### Antiproliferative Activity

The antiproliferative properties of synthesized compounds are essential in assessing their therapeutic potential. In this study, we embarked on a detailed evaluation of compounds **7**–**21**, focusing on their ability to hinder cell proliferation across diverse cancer cell lines. The chosen cell lines represent a spectrum of human cancers, which are pancreatic cancer (Panc-1), breast cancer (MCF-7), colon cancer (HT-29), and epithelial cancer (A-549). This variety ensures that the synthesized compounds’ efficacy is tested against different cancer types, giving a holistic picture of their potential use. For a rigorous assessment, the widely accepted MTT assay [63,64] was employed. Using Doxorubicin, a well-established drug, as the reference standard ensures a benchmark for the synthesized compounds, allowing a contextual understanding of their efficacy. A first glance at the results in Table 2 showcases the promising antiproliferative potential of the synthesized compounds. With GI_50_ values spanning 1.40 µM to 9.40 µM, these compounds exhibited commendable inhibitory effects compared to the potent Doxorubicin with a GI_50_ of 1.10 µM. Among the series, compounds **8**, **9**, and **18**–**21** emerged as the frontrunners. Their antiproliferative activity was strikingly high, especially in compound **18** with a GI_50_ value tantamount to Doxorubicin, thereby indicating its impressive potency. The antiproliferative activity seems to hinge significantly on the type of substituent at the N-3 position of the quinazoline moiety. Compound **18**, with an allyl group, showed a pronounced effect, whereas its counterparts with ethyl or phenyl groups, i.e., compounds **8** and **13**, were less potent. This suggests the paramount role of allyl group in enhancing the antiproliferative activity, a crucial insight for future design improvements. Diving deeper into the structure–activity relationship, the position-4 substitution on the phenyl group of the pyridine-2-one moiety emerges as a critical determinant. Compounds **19**, **20**, and **21**, bearing halogens, suggest a clear tolerance and even preference for halogens in antiproliferative activity. Interestingly, the order of efficacy based on the halogens is Cl > Br > F. Unsubstituted derivatives, namely compounds **7**, **12**, and **17**, showcased relatively lower potency. This signifies the relevance of the substituents at position-4 of the phenyl group, stressing their importance in future design considerations.

In synthesizing compounds for antiproliferative purposes, the interplay between different functional groups and substituents becomes apparent. This detailed evaluation of the synthesized compounds offers valuable insights, not just in understanding their current efficacy but also in directing future compound designs and modifications for even enhanced therapeutic potential.

##### EGFR Inhibitory Activity

The most effective antiproliferative hybrids, **8**, **9**, **18**, and **19**, were tested for EGFR-TK inhibitory activity as a possible target for their antiproliferative activity [65]. Table 3 shows the IC_50_ values for each compound against Erlotinib, the reference drug.

Compounds **8**, **9**, **18**, and **19** displayed promising EGFR inhibitory activity, with IC_50_ values ranging from 110 nM to 190 nM compared to Erlotinib (IC_50_ = 80 ± 5 nM). As EGFR inhibitors, the investigated compounds were less effective than Erlotinib. The most potent antiproliferative agent, compound **18** (R_1_ = allyl, R_2_ = OCH_3_), was also the most potent EGFR inhibitor, with an IC_50_ value of 110 ± 10 nM, 1.4-fold less potent than Erlotinib. Compound **19** (R_1_ = allyl, R_2_ = Cl) was the second most active compound, with an IC_50_ value of 140 ± 11 nM, 1.8-fold less potent than Erlotinib. These findings add to the body of evidence supporting the significance of the methoxy group in these compounds’ antiproliferative effect. Finally, compounds **8** (R_1_ = ethyl, R_2_ = OCH_3_) and **9** (R_1_ = ethyl, R_2_ = Cl) were the least effective EGFR inhibitors, with IC_50_ values of 160 ± 12 nM and 190 ± 15 nM, respectively. These findings indicate that the EGFR may be a potential target for the antiproliferative activity of the investigated compounds, notably compounds **18** and **19**.

##### BRAF^V600E^ Inhibitory Activity

An in vitro study was carried out to evaluate the activity of the newly synthesized compounds **8**, **9**, **18**, and **19** against BRAF^V600E^ [59]. Table 3 shows the IC_50_ values for the tested compounds and Erlotinib, which was chosen as the reference drug. Table 2 shows that the investigated compounds had good inhibitory efficacy, with IC_50_ values ranging from 140 nM to 290 nM compared to Erlotinib (IC_50_ = 60 ± 5 nM). Compounds **18** (R_1_ = allyl, R_2_ = OCH_3_) and **19** (R_1_ = allyl, R_2_ = Cl) had the most potent inhibitory activities against BRAF^V600E^, with IC_50_ values of 140 ± 10 nM and 190 ± 12 nM, respectively, being 2- and 3-fold less potent than Erlotinib. According to the findings of this study, the tested compounds **18** and **19** had dual inhibitory effects against both EGFR and BRAF^V600E^, suggesting that they could be used as potential targets for antiproliferative action.

### 2.3. In Silico Studies

#### 2.3.1. Docking Study

In this investigative study, we conducted a computational docking analysis to unveil the binding interactions involving the highly in vitro active compounds **18** and **19** with the tyrosine kinase receptors EGFR and BRAF^V600E^. We employed the Discovery Studio software, a computational tool designed for such analyses [66,67,68]. To streamline our inquiry, we obtained the X-ray crystallography structures of the EGFR and BRAF^V600E^ tyrosine kinases. Specifically, the structure of EGFR was co-crystallized with Erlotinib as a reference structure (PDB: 1M17) [69]. Similarly, the BRAF^V600E^ structure was co-crystallized with Vemurafenib as a reference (PDB: 3OG7) [70].

**Figure 4:**
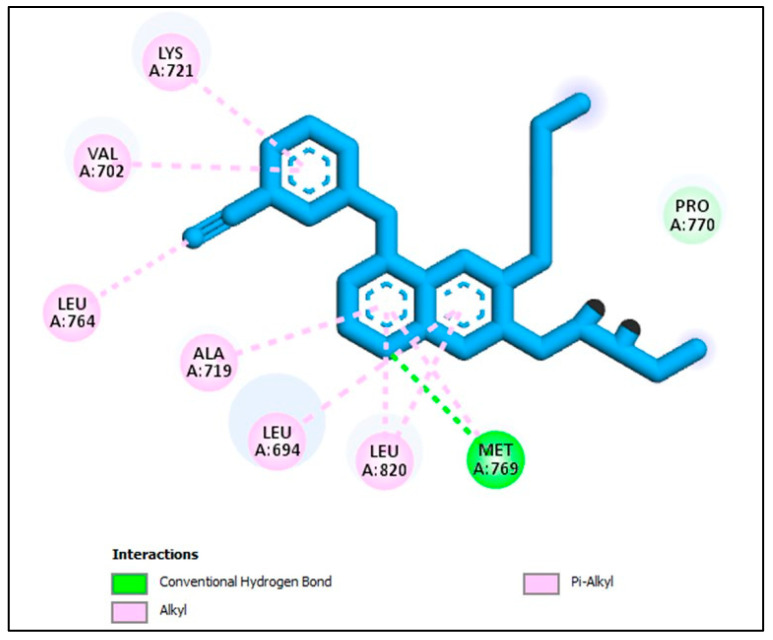
Docking representation model of co-crystallized Erlotinib within the binding site of EGFR (PDB ID: 1M17).

To validate the reliability of the EGFR docking procedure, we conducted a re-docking experiment using the co-crystallized Erlotinib. This was performed within the active site of EGFR, resulting in a S score of −8.69 kcal/mol. The root-mean-square deviation (RMSD) value, a structural similarity measure, was calculated to be 1.72 Å. A significant hydrogen bond interaction was established, specifically between the pyrimidine nitrogen of Erlotinib and the amino acid Met769, with a bond length of 2.29 Å. This interaction emphasized the pivotal role of Met769 in stabilizing the ligand within the binding site Figure 4.

**Figure 5:**
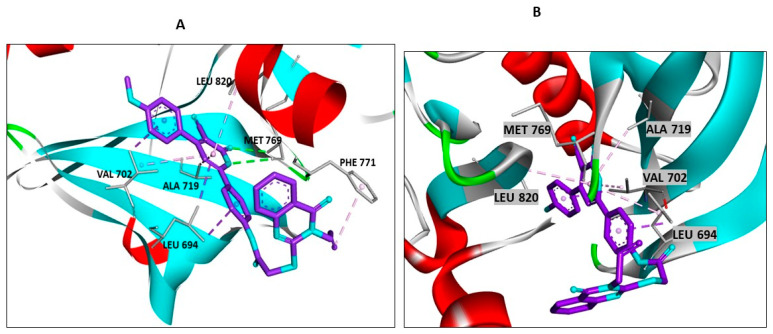
Docking representation models of compounds **18** and **19** within the binding site of EGFR (PDB ID: **1M17**); (**A**) 3D-docked model of compound **18**; (**B**) 3D-docked model of compound **19**.

**Figure 6:**
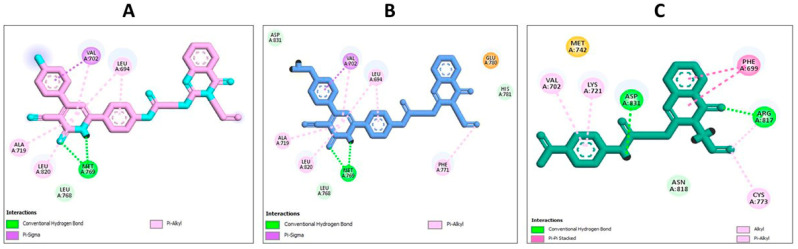
Docking representation models of compound **18**, **19**, and **5c** within the binding site of EGFR (PDB ID: **1M17**); (**A**) 2D-docked model of compound **19**; (**B**) 2D-docked model of compound **18**; (**C**) 2D-docked model of compound **5c**.

The molecular modelling and interactions presented in the docking studies of compounds **18**, **19**, and the quinazoline-single moiety 5C with the EGFR receptor provide a profound insight into the mechanisms and intricacies of drug-receptor binding. The conjugation of quinazolin-4-one and 3-cyanopyridin-2-one motifs into a singular entity in compounds **18** and **19** and their subsequent interactions illuminate the significance of strategic molecular design for targeted receptor engagement. A critical observation from the provided data is the pronounced binding efficacy of the conjugated hybrids, **18** and **19**, despite the evident absence of direct interactions of their quinazoline ring with the receptor amino acids. This absence might initially seem counterintuitive, given the potent nature of quinazoline as a pharmacophore. However, the enhanced activity of these hybrids can be attributed to their encompassing and vital interactions with pivotal EGFR amino acids. Such interactions not only anchor the molecules within the active site, but they also likely disrupt native functions of the receptor, rendering it inactive (Figure 5). The diversity and multiplicity of the interactions exhibited by compounds **18** and **19** emphasize the wisdom behind the conjugation of the two moieties. Whereas each moiety is capable of specific interactions, their combination seems to exploit a broader range of binding possibilities, ensuring a comprehensive engagement with the receptor. This intricate binding landscape, especially the interactions with crucial amino acids like MET A:769, ALA A:719, and VAL A:702, indicates a highly strategic alignment within the receptor active site, which could explain their potent in vitro activity. Compound **18** establishes a more diverse binding pattern within the active site of EGFR compared to compound **19**. This diversity is evident in its broader interaction landscape, especially its π-sigma and π-alkyl interactions with pivotal amino acids like PHE A:771 Figure 5. These extensive interactions not only stabilize the compound within the active site but could also enhance its inhibitory effect on the receptor. Furthermore, compound **18**′s computed docking score of −7.35 kcal/mol, which is higher than the −6.85 kcal/mol score of compound **19**, provides a quantitative measure of its superior binding affinity (Figure 5). The constrained binding pattern of compound **19**, in contrast, might limit its binding stability and, consequently, its therapeutic efficacy. Whereas compound **19** still exhibits valuable interactions and remains a potential EGFR inhibitor, compound **18**, with its multifaceted engagement and superior docking score, stands out as the more potent entity in the series. On the contrary, the quinazoline-single moiety **5c**, although demonstrating a series of interactions, lacks the diversity and depth of engagements witnessed in the hybrids (Figure 6). This difference is likely due to the absence of the 3-cyanopyridin-2-one motif, which, when present in the hybrids, amplifies their binding profiles. The lower S score for **5c** compared to the hybrids further supports this notion (Figure 6).

**Figure 7:**
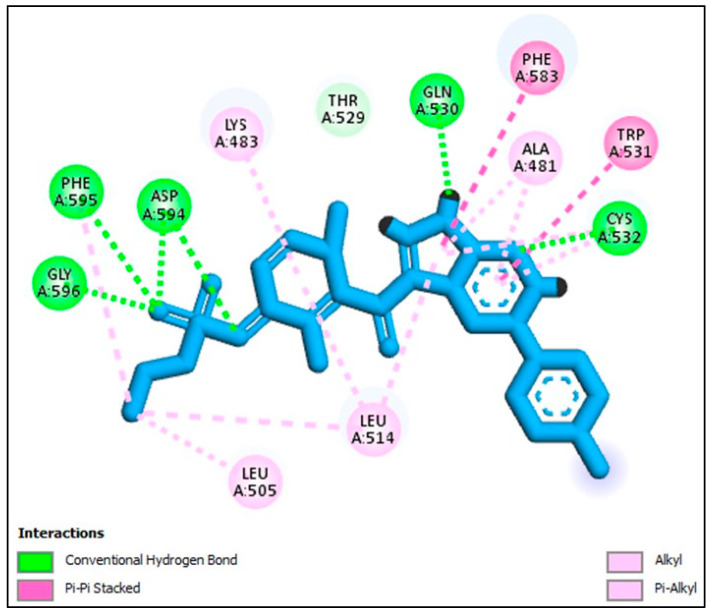
Docking representation model of co-crystallized Vemurafenib within the binding site of BRAF (PDB ID: 3OG7).

Similarly, to validate the docking protocol concerning BRAF^V600E^, we conducted a re-docking experiment utilizing the co-crystallized Vemurafenib. This endeavor took place at active site of BRAF^V600E^, resulting in an impressive S score of −11.99 kcal/mol. To gauge the extent of structural conformity, we computed a root-mean-square deviation (RMSD) value of 1.55 Å. Substantial hydrogen bond interactions were established, particularly between Vemurafenib and the amino acids Cys532 and Asn580, firmly anchoring its position within the binding site (Figure 7).

**Figure 8:**
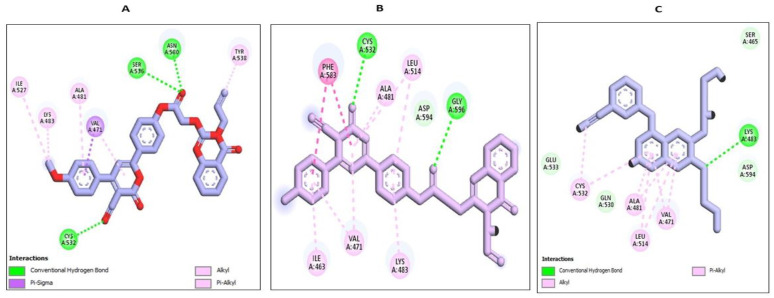
Docking representation models of compound **18**, **19**, and Erlotinib within the binding site of BRAF (PDB ID: 3OG7); (**A**) 2D-docked model of compound **18**; (**B**) 2D-docked model of compound **19**; (**C**) 2D-docked model of compound Erlotinib.

Hybrid **18**, with a docking score of −7.85 kcal/mol, showcases more pronounced interactions with the receptor. From the visualization, hybrid **18** forms strong conventional hydrogen bonds with residues like CYS A:532, ASN A:580, and SER A:536 (Figure 8). These interactions, particularly with CYS A:532, are of utmost importance, considering the pivotal role cysteine residues often play in the active sites of various proteins. The compound aromatic regions also engage in multiple pi interactions, further reinforcing its grip within the active site. Alkyl and pi-alkyl interactions with residues such as ALA A:481, LYS A:483, and VAL A:471 solidify its binding affinity, potentially leading to its potent in vitro activity (Figure 8). On the other hand, hybrid **19**, despite having a commendable docking score of −7.53 kcal/mol, falls slightly short when compared to hybrid **18**. The visual representation suggests robust interactions, but the comparative difference in docking scores indicates that hybrid **18** might have a slightly superior binding affinity or forms more favorable interactions, translating to enhanced inhibitory potential. However, with a docking score of −6.33 kcal/mol, Erlotinib interaction is somewhat less optimal than the scores observed for hybrids **18** and **19** (Figure 8). One reason for this could be the extent and depth of interactions Erlotinib forms with the receptor. Whereas it does form important bonds with vital residues, it may not span as diverse a landscape as the hybrids, especially hybrid **18** (Figure 8). These findings closely aligned with dual EGFR/BRAF^V600E^ in vitro inhibition assay outcomes. In conclusion, the findings presented in this study underscore the notable inhibitory potential of compound **18** against the BRAF^V600E^ kinase. This propensity is underpinned by meaningful binding interactions observed within the active site. This observation resonates with the outcomes obtained from the rigorous BRAF^V600E^ in vitro inhibition assay, lending credibility to the predictive capabilities of our computational approach.

Additionally, our investigation unveils a compelling avenue for potential dual inhibition, as indicated by supplementary simulations targeting both EGFR and BRAF^V600E^; specifically, compounds **18** and **19** promise to function as dual inhibitors.

The insights garnered from this study offer a foundational comprehension of the inhibitory implications embedded within the investigated compounds. This understanding, in turn, sets the stage for subsequent experimental investigations and optimization endeavors within the dynamic realm of drug discovery. Describing the complex interactions between these compounds and the target kinases is an important guide for developing targeted hit compounds. These compounds could interfere with pathways linked to cancer signaling.

#### 2.3.2. In Silico ADMET Studies

Considering the promising in vitro and silico docking results, we proceeded to conduct supplementary ADMET studies for the newly synthesized compounds. This choice was motivated by the intention to gain a more comprehensive insight into these significant activities [71]. During the ADMET investigations, we utilized Erlotinib as the established reference compound. Using Discovery Studio 4.0, we conducted predictions for the ADMET descriptors of all synthesized compounds. The expected descriptors are outlined in Table 4.

A significant number of the hybrid compounds exhibited a modest predicted level of intestinal absorption (absorption level = 2). This positioning designates them as promising candidates for localized treatment of gastrointestinal tumors or as potential contenders for intravenous administration. Notably, most of these novel compounds displayed low solubility (ADME aqueous solubility level = 2).

Furthermore, the AlogP98 tool aided in predicting the logP value based on the molecular structure of each compound. This approach allows researchers to estimate how a compound will partition between a nonpolar solvent (octanol) and water. Notably, most hybrid compounds demonstrated AlogP98 values ranging from 5 to 6, indicating their heightened lipophilicity. This characteristic contributes to their reduced aqueous solubility. However, there is a potential solution in the form of Cyclodextrin Complexation, a method that improves solubility by encapsulating these hybrid molecules within the hydrophobic cavity of cyclodextrin molecules.

During the ADMET assessment, all the newly synthesized hybrids were categorized at a blood–brain barrier (BBB) level of 4, effectively hindering their ability to penetrate across the BBB. Importantly, the drug bioavailability was interconnected with the fundamental property of 2D polar surface area (ADMET 2D PSA).

**Figure 9:**
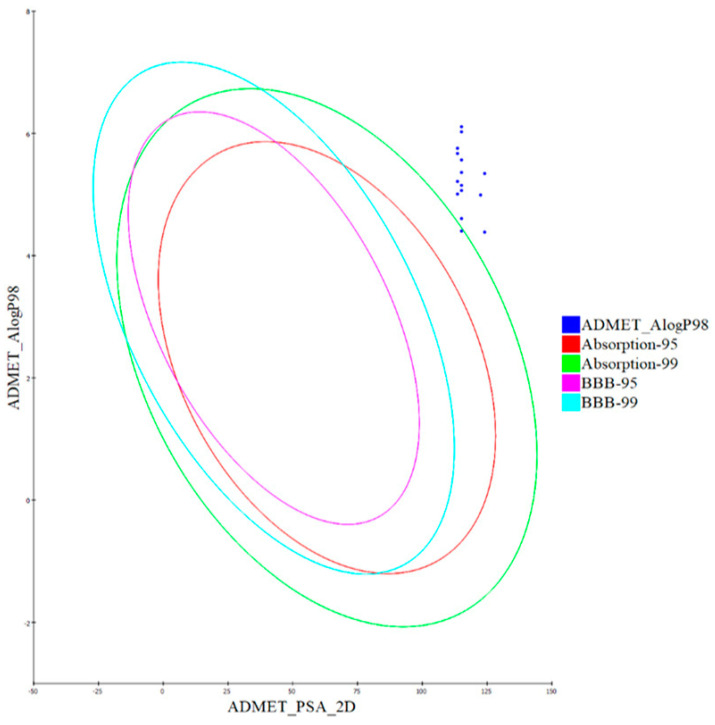
The expected ADMET study of quinazolin-4-one/3-cyanopyrid-2-one hybrids **7**–**21**.

Utilizing the calculated 2D polar surface area (PSA 2D) and Atom-based Log P98 (A log P98) properties, the outcomes have been visualized through a 2D ADMET plot (Figure 9). It is worth noting that molecules with a PSA less than 145 generally exhibit characteristics of low bioavailability and passive absorption [72]. Using a 2D chemical structure as input, the cytochrome P450 2D6 (CYP2D6) model predicts the potential for inhibiting the CYP2D6 enzyme. This enzyme, located in the liver, plays a critical role in metabolizing a wide range of substrates, thereby significantly contributing to various drug–drug interaction scenarios [73]. As a result, experimenting to evaluate CYP2D6 inhibition becomes crucial within the regulatory protocols followed during drug discovery and development [74]. Each evaluated hybrid compound was predicted to display non-inhibitory behavior towards CYP2D6. As a result, the risk of inducing liver dysfunction after administering these hybrids is minimal.

The plasma protein binding model determines whether a substance will bind robustly (>90%) to carrier proteins in the blood. Notably, a substantial binding to plasma proteins (>90%) was expected for the majority of hybrid compounds, as detailed in Table 4.

#### 2.3.3. In Silico Toxicity Predictions

To predict toxicity, we utilized the constructed and validated models within the Discovery Studio software for the synthesized compounds [75]. The rodent carcinogenicity test carried out by the FDA evaluates the capacity of a chemical structure to induce cancer in rats. On the other hand, the prediction of rat maximum tolerated dose (MTD) estimates the highest dose of a chemical substance that can be administered to rats without resulting in adverse effects [76]. In assessing toxicity for a chemical compound, the prediction of rat oral LD_50_ is utilized to forecast the acute median lethal dose (LD_50_) in rats after the compound is administered orally [77]. As part of the Draize test, ocular irritancy analysis is utilized to determine the potential of a particular compound to cause eye irritation and assess the degree of severity of this irritation [78]. In evaluations based on rabbits, investigations into skin irritancy assess the probability of a substance causing skin irritation and the potential severity of such effects. As per the in silico assessments outlined in Table 5, most compounds displayed low toxicity and minimal adverse effects.

Additionally, all the hybrid compounds subjected to testing were forecasted to have non-carcinogenic attributes, aligning with the preliminary findings of the FDA rodent carcinogenicity assessment. The majority of the evaluated compounds exhibited higher rat oral LD50 values compared to the value of Erlotinib.

Furthermore, the predictive models suggested that all hybrids are anticipated to not irritate in the context of skin irritancy testing, and the majority of them are projected to induce mild irritation in the context of ocular irritancy testing.

In conclusion, the ADMET investigations conducted in this study provide crucial insights into the potential effectiveness, safety, and pharmacokinetic behavior of the newly developed hybrids. The insights gained from these assessments hold immense significance in guiding the drug discovery and development trajectory. This wealth of information assists in identifying promising drug candidates that merit further evaluation and advancement in the testing and developmental stages.

In summation, the ADMET explorations undertaken in this investigation offer pivotal insights into the potential efficacy, safety profile, and pharmacokinetic attributes of the recently formulated hybrids. The discernments gleaned from these analyses carry substantial importance, serving as a compass to navigate the path of drug discovery and development. This reservoir of knowledge aids in pinpointing auspicious drug candidates warranting meticulous scrutiny and progression through subsequent testing and developmental phases.

### 2.4. Structure–Activity Relationship (SAR)

Based on the observed results, the structure–activity relationship of our novel quinazolin-4-one/3-cyanopyridin-2-one hybrids (**7**–**21**) is as follows.

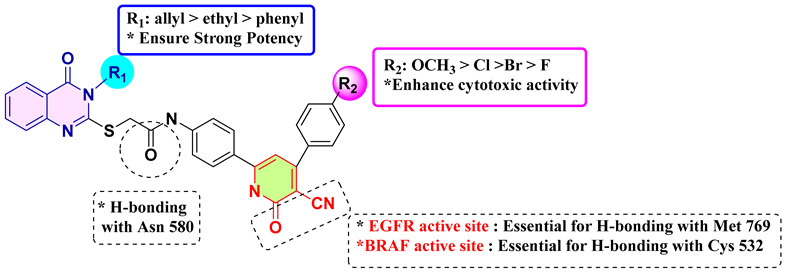



Finally, introducing substituents into the quinazolin-4-one and 3-cyanopyridin-2-one moieties significantly influences the efficacy of the newly synthesized hybrids **7**–**21**. The highest levels of anti-proliferative activity were observed in 3-allyl quinazolin-4-one derivatives, with 3-ethyl and 3-phenyl derivatives displaying lower levels of activity in that order. The addition of specific functional groups, such as an electron-donating methoxy group at position 4 of the phenyl group in the 3-cyano-2-pyridone moiety (as seen in compound **18**), appears to enhance both EGFR and BRAF inhibitory activity. The introduction of electron-withdrawing substituents at the para position of phenyl at position 4 of 3-cyano-2-pyridone seems to improve cytotoxicity in the order (4-Cl > 4-Br > 4-F). Moreover, the presence of a nitrile group (CN) at the C3 and carbonyl group of 3-cyano-2-pyridone moiety are essential for binding EGFR and BRAF active site. Moreover, both the pyridone nitrogen and the carbonyl oxygen atom within the 3-cyanopyrid-2-one core form hydrogen bonds with the backbone NH of Met769 located in the hinge region of the active site of EGFR kinase. On other hand, the nitrile group within the 3-cyanopyrid-2-one core is essential for forming a hydrogen bond with the key residue Cys 532 in the N-lobe, while the carbonyl oxygen atom of the amidic linkage is crucial for creating a hydrogen bond with Asn 580 within the catalytic loop active site of BRAF^V600E^.The strategic positioning of these functional groups is critical in defining these compounds’ antiproliferative efficacy.

## 3. Materials and Methods

### 3.1. Chemistry

General details: see Section A (Supplementary File). Compounds **3a**–**c** and **5a**–**c** were prepared according to previous studies [33,35].

#### General Procedures for the Synthesis of Compounds **7**–**21**

A mixture of appropriate derivatives **5a**–**c** (1 mmol) substituted benzaldehydes **6a**–**j** (1 mmol), ethyl cyanoacetate (0.113 g, 1 mmol), and ammonium acetate (0.154 g, 2 mmol) were fused at 120–130 °C for 30 min. After cooling, the crude product was filtered off and crystallized from absolute ethanol [48,52].

*N-(4-(5-Cyano-6-oxo-4-phenyl-1,6-dihydropyridin-2-yl)phenyl)-2-((3-ethyl-4-oxo-3,4-dihydroquinazolin-2-yl)thio)acetamide* (**7**). White powder; yield (0.400 g, 75%); m.p.: > 300 °C; ^1^H NMR (500 MHz, DMSO-*d*_6_) δ (ppm): 1.33 (t, *J* = 7.0 Hz, 3H, N3-CH_2_-CH_3_), 4.14 (q, *J* = 7.1 Hz, 2H, N3-CH_2_-CH_3_), 4.27 (s, 2H, SCH_2_), 6.74 (s, 1H, pyridine-C_5_–H), 7.41–7.48 (m, 4H, Ar-H), 7.57–7.60 (m, 3H, Ar-H), 7.77 (d, *J* = 8.2 Hz, 3H, Ar-H), 7.92 (d, *J* = 8.5 Hz, 2H, Ar-H), 8.08 (d, *J* = 7.9 Hz, 1H, Ar-H), 10.73 (s, 1H, CONH), 12.67 (br s, 1H, pyridine-NH). ^13^C NMR (125 MHz, DMSO-d_6_) δ (ppm): 13.02, 36.78, 37.61, 91.52, 104.39, 118.53, 118.83, 118.88, 121.61, 124.00, 125.67, 125.99, 126.43, 127.50, 128.69, 130.37, 131.29, 131.77, 134.68, 135.36, 138.26, 141.76, 146.68, 155.88, 160.21, 166.36. Anal. Calcd. For C_30_H_23_N_5_O_3_S (533.61): C, 67.53; H, 4.34; N, 13.12; S, 6.01. Found: C, 67.64; H, 4.46; N, 13.15; S, 6.23.
*N-(4-(5-Cyano-4-(4-methoxyphenyl)-6-oxo-1,6-dihydropyridin-2-yl)phenyl)-2-((3-ethyl-4-oxo-3,4-dihydroquinazolin-2-yl)thio)acetamide* (**8**). Yellow powder; yield (0.292 g, 80%); m.p.: > 300 °C; ^1^H NMR (500 MHz, DMSO-*d*_6_) δ (ppm): 1.33 (t, *J* = 7.0 Hz, 3H, N3-CH_2_-CH_3_), 3.85 (s, 3H, OCH_3_), 4.14 (q, *J* = 7.1 Hz, 2H, N3-CH_2_-CH_3_), 4.27 (s, 2H, SCH_2_), 6.74 (s, 1H, pyridine-C_5_–H), 7.10–7.16 (m, 2H, Ar-H), 7.40–7.46 (m, 2H, Ar-H), 7.72–7.78 (m, 5H, Ar-H), 7.89 (s, 2H, Ar-H), 8.07 (dd, *J* = 8.2, 1.6 Hz, 1H, Ar-H), 10.73 (s, 1H, CONH), 12.61 (br s, 1H, pyridine-NH). ^13^C NMR (125 MHz, DMSO-*d*_6_) δ (ppm): 13.02, 36.78, 37.61, 55.61, 91.52, 104.39, 118.53, 118.83, 118.88, 121.61, 124.00, 125.67, 125.99, 126.43, 127.50, 128.69, 130.37, 131.29, 131.77, 134.68, 135.36, 138.26, 141.76, 146.68, 155.88, 160.21, 166.36. Anal. Calcd. For C_31_H_25_N_5_O_4_S (563.63): C, 66.06; H, 4.47; N, 12.43; S, 5.69. Found: C, 66.28; H, 4.67; N, 12.44; S, 5.71.
*N-(4-(4-(4-Chlorophenyl)-5-cyano-6-oxo-1,6-dihydropyridin-2-yl)phenyl)-2-((3-ethyl-4-oxo-3,4-dihydroquinazolin-2-yl)thio)acetamide* (**9**). Yellow powder; yield (0.282 g, 78%); m.p.: > 300 °C; ^1^H NMR (500 MHz, DMSO-*d*_6_) δ (ppm): 1.33 (t, *J* = 7.0 Hz, 3H, N3-CH_2_-CH_3_), 4.13 (q, *J* = 7.1 Hz, 2H, N3-CH_2_-CH_3_), 4.26 (s, 2H, SCH_2_), 6.77 (s, 1H, pyridine-C_5_–H), 7.34–7.48 (m, 3H, Ar-H), 7.58–7.71 (m, 3H, Ar-H), 7.72–7.85 (m, 5H, Ar-H), 8.04 –8.09 (m, 1H, Ar-H), 10.73 (s, 1H, CONH), 12.75 (s, 1H, pyridine-NH). ^13^C NMR (125 MHz, DMSO-d_6_) δ (ppm): 13.02, 36.78, 38.01, 91.52, 104.45, 115.26, 118.83, 118.87, 125.67, 125.99, 126.43, 127.50, 128.69, 130.37, 131.29, 131.77, 134.68, 135.36, 141.76, 146.68, 152.76, 155.88, 157.34,158.88, 159.35, 160.21, 166.36. Anal. Calcd. For C_30_H_22_ClN_5_O_3_S (568.05): C, 63.43; H, 3.90; N, 12.33; S, 5.64. Found: C, 63.45; H, 4.10; N, 12.35; S, 5.66.
*N-(4-(4-(4-Bromophenyl)-5-cyano-6-oxo-1,6-dihydropyridin-2-yl)phenyl)-2-((3-ethyl-4-oxo-3,4-dihydroquinazolin-2-yl)thio)acetamide* (**10**). Yellow powder; yield (0.262 g, 78%); m.p.: > 300 °C; ^1^H NMR (500 MHz, DMSO-*d*_6_) δ (ppm): 1.33 (t, *J* = 7.0 Hz, 3H, N3-CH_2_-CH_3_), 4.13 (q, *J* = 7.1 Hz, 2H, N3-CH_2_-CH_3_), 4.26 (s, 2H, SCH_2_), 6.77 (s, 1H, pyridine-C_5_–H), 7.34–7.48 (m, 3H, Ar-H), 7.58–7.71 (m, 3H, Ar-H), 7.72–7.85 (m, 5H, Ar-H), 8.04–8.09 (m, 1H, Ar-H), 10.73 (s, 1H, CONH), 12.75 (s, 1H, pyridine-NH). ^13^C NMR (125 MHz, DMSO*-d*_6_) δ (ppm): 13.02, 36.78, 38.01, 91.52, 104.45, 115.26, 118.83, 118.87, 125.67, 125.99, 126.43, 127.50, 128.69, 130.37, 131.29, 131.77, 134.68, 135.36, 141.76, 146.68, 152.76, 155.88, 157.34, 158.88, 159.35, 160.21, 166.36. Anal. Calcd. For C_30_H_22_BrN_5_O_3_S (612.50): C, 58.83; H, 3.62; N, 11.43; S, 5.23. Found: C, 58.90; H, 3.68; N, 11.48; S, 5.28.
*N-(4-(5-Cyano-4-(4-fluorophenyl)-6-oxo-1,6-dihydropyridin-2-yl)phenyl)-2-((3-ethyl-4-oxo-3,4-dihydroquinazolin-2-yl)thio)acetamide* (**11**). Yellow powder; yield (0.298 g, 80%); m.p.: > 300 °C; ^1^H NMR (500 MHz, DMSO-d_6_) δ (ppm): 1.33 (t, *J* = 7.0 Hz, 3H, N3-CH_2_-CH_3_), 4.13 (q, *J* = 7.1 Hz, 2H, N3-CH_2_-CH_3_), 4.26 (s, 2H, SCH_2_), 6.77 (s, 1H, pyridine-C_5_–H), 7.34–7.48 (m, 3H, Ar-H), 7.58–7.71 (m, 3H, Ar-H), 7.72–7.85 (m, 5H, Ar-H), 8.04–8.09 (m, 1H, Ar-H), 10.73 (s, 1H, CONH), 12.75 (s, 1H, pyridine-NH). ^13^C NMR (125 MHz, DMSO*-d*_6_) δ (ppm): 13.02, 36.78, 38.01, 91.52, 104.45, 115.26, 118.83, 118.87, 125.67, 125.99, 126.43, 127.50, 128.69, 130.37, 131.29, 131.77, 134.68, 135.36, 141.76, 146.68, 152.76, 155.88, 157.34, 158.88, 159.35, 160.21, 166.36. Anal. Calcd. For C_30_H_22_FN_5_O_3_S (551.60): C, 65.32; H, 4.02; N, 12.70; S, 5.81. Found: C, 65.42; H, 4.22; N, 12.60; S, 5.85.
*N-(4-(5-Cyano-6-oxo-4-phenyl-1,6-dihydropyridin-2-yl)phenyl)-2-((4-oxo-3-phenyl-3,4-dihydroquinazolin-2-yl)thio)acetamide* (**12**). Yellow powder; yield (0.298 g, 80%); m.p.: > 300 °C; ^1^H NMR (500 MHz, DMSO*-d*_6_) δ: 4.11 (s, 2H, SCH_2_), 6.78 (s, 1H, pyridine-C_5_–H), 7.43–7.48 (m, 1H, Ar-H), 7.48–7.51 (m, 2H, Ar-H), 7.51–7.56 (m, 2H, Ar-H), 7.56–7.58 (m, 2H, Ar-H), 7.59–7.62 (m, 3H, Ar-H), 7.70–7.76 (m, 4H, Ar-H), 7.78–7.84 (m, 1H, Ar-H), 7.90 (d, *J* = 8.3 Hz, 2H, Ar-H), 8.08 (m, 1H, Ar-H), 10.67 (s, 1H, CONH), 12.71 (s, 1H, pyridine-NH). ^13^C NMR (125 MHz, DMSO*-d*_6_) δ (ppm): 37.43, 91.84, 104.34, 108.15, 116.84, 119.18, 119.67, 126.10, 126.38, 126.85, 128.39, 128.86, 129.04, 129.57, 129.85, 130.32, 130.66, 135.25, 135.90, 136.30, 138.07, 141.85, 143.68, 147.28, 157.06, 158.78, 160.90, 166.71. Anal. Calcd. For C_34_H_23_N_5_O_3_S (581.65): C, 70.21; H, 3.99; N, 12.04; S, 5.51. Found: C, 70.41; H, 3.95; N, 12.01; S, 5.50.
*N-(4-(5-Cyano-4-(4-methoxyphenyl)-6-oxo-1,6-dihydropyridin-2-yl)phenyl)-2-((4-oxo-3-phenyl-3,4-dihydroquinazolin-2-yl)thio)acetamide* (**13**). Yellow powder; yield (0.332 g, 80%); m.p.: > 300 °C; ^1^H NMR (500 MHz, DMSO*-d*_6_) δ (ppm): 3.86 (s, 3H, OCH_3_), 4.13 (s, 2H, SCH_2_), 6.78 (s, 1H, pyridine-C_5_–H), 7.13 (d, *J* = 8.4 Hz, 2H, Ar-H), 7.44–7.58 (m, 4H, Ar-H), 7.62 (d, *J* = 5.7 Hz, 3H, Ar-H), 7.66–7.85 (m, 5H, Ar-H), 7.90 (d, *J* = 8.3 Hz, 2H, Ar-H), 8.09 (d, *J* = 7.9 Hz, 1H, Ar-H), 10.68 (s, 1H, CONH), 12.58 (br s, 1H, pyridine-NH). ^13^C NMR (125 MHz, DMSO-*d*_6_) δ (ppm): 36.81, 55.22, 91.02, 112.65, 115.63, 118.17, 119.34, 120.04, 122.73, 123.64, 126.34, 126.55, 127.13, 129.17, 129.90, 130.05, 130.52, 130.85, 132.25, 135.44, 135.79, 136.25, 142.22, 147.58, 157.39, 160.59, 165.72. Anal. Calcd. For C_35_H_25_N_5_O_4_S (611.68): C, 68.73; H, 4.12; N, 11.45; S, 5.24. Found: C, 68.75; H, 4.17; N, 11.50; S, 5.25.
*N-(4-(4-(4-Chlorophenyl)-5-cyano-6-oxo-1,6-dihydropyridin-2-yl)phenyl)-2-((4-oxo-3-phenyl-3,4-dihydroquinazolin-2-yl)thio)acetamide* (**14**). Yellow powder; yield (0.309 g, 75%); m.p.: > 300 °C; ^1^H NMR (500 MHz, DMSO-*d*_6_) δ (ppm): 4.13 (s, 2H, SCH_2_), 6.82 (s, 1H, pyridine-C_5_–H), 7.47 (d, *J* = 7.5 Hz, 1H, Ar-H), 7.49–7.52 (m, 2H, Ar-H), 7.54 (d, *J* = 8.0 Hz, 1H, Ar-H), 7.57–7.63 (m, 3H, Ar-H), 7.63–7.68 (m, 2H, Ar-H), 7.72–7.79 (m, 4H, Ar-H), 7.79–7.84 (m, 1H, Ar-H), 7.91 (d, *J* = 8.3 Hz, 2H, Ar-H), 8.09 (dd, *J* = 8.0, 1.6 Hz, 1H, Ar-H), 10.68 (s, 1H, CONH), 12.76 (br s, 1H, pyridine-NH). ^13^C NMR (125 MHz, DMSO-*d*_6_) δ (ppm): 37.82, 91.02, 104.65, 119.34, 120.04, 122.73, 123.64, 124.51, 126.34, 126.55, 127.13, 129.17, 129.90, 130.05, 130.52, 130.85, 132.25, 135.44, 135.79, 136.25, 142.22, 147.58, 157.39, 161.08, 166.86. Anal. Calcd. For C_34_H_22_ClN_5_O_3_S (616.09): C, 66.28; H, 3.60; N, 11.37; S, 5.20. Found: C, 66.30; H, 3.70; N, 11.36; S, 5.29.
*N-(4-(4-(4-Bromophenyl)-5-cyano-6-oxo-1,6-dihydropyridin-2-yl)phenyl)-2-((4-oxo-3-phenyl-3,4-dihydroquinazolin-2-yl)thio)acetamide* (**15**). Yellow powder; yield (0.296 g, 77%); m.p.: > 300 °C; ^1^H NMR (500 MHz, DMSO*-d*_6_) δ (ppm): 4.13 (s, 2H, SCH_2_), 6.80 (s, 1H, pyridine-C_5_–H), 7.47 (d, *J* = 7.5 Hz, 1H, Ar-H), 7.49–7.52 (m, 2H, Ar-H), 7.54 (d, *J* = 8.0 Hz, 1H, Ar-H), 7.57–7.63 (m, 3H, Ar-H), 7.63–7.68 (m, 2H, Ar-H), 7.72–7.79 (m, 4H, Ar-H), 7.79–7.84 (m, 1H, Ar-H), 7.91 (d, *J* = 8.3 Hz, 2H, Ar-H), 8.09 (dd, *J* = 8.0, 1.6 Hz, 1H, Ar-H), 10.68 (s, 1H, CONH), 12.76 (br s, 1H, pyridine-NH). ^13^C NMR (125 MHz, DMSO-*d*_6_) δ (ppm): 37.82, 91.02, 104.65, 119.34, 120.04, 122.73, 123.64, 124.51, 126.34, 126.55, 127.13, 129.17, 129.90, 130.05, 130.52, 130.85, 132.25, 135.44, 135.79, 136.25, 142.22, 147.58, 157.39, 161.08, 166.86. Anal. Calcd. For C_34_H_22_BrN_5_O_3_S (660.55): C, 61.82; H, 12.10; N, 7.27; S, 4.85. Found: C, 61.84; H, 12.30; N, 7.47; S, 4.90.
*N-(4-(5-Cyano-4-(4-fluorophenyl)-6-oxo-1,6-dihydropyridin-2-yl)phenyl)-2-((4-oxo-3-phenyl-3,4-dihydroquinazolin-2-yl)thio)acetamide* (**16**). Yellow powder; yield (0.339 g, 80%); m.p.: > 300 °C; ^1^H NMR (500 MHz, DMSO-*d*_6_) δ 4.11 (s, 2H, SCH_2_), 6.80 (s, 1H, pyridine-C_5_–H), 7.17–7.34 (m, 1H, Ar-H), 7.34–7.56 (m, 5H, Ar-H), 7.56 –7.68 (m, 3H, Ar-H), 7.68–7.77 (m, 3H, Ar-H), 7.77–7.84 (m, 2H, Ar-H), 7.85–8.02 (m, 2H, Ar-H), 8.08 (dd, *J* = 8.0, 1.6 Hz, 1H), 10.67 (s, 1H, CONH), 12.71 (br s, 1H, pyridine-NH). ^13^C NMR (125 MHz, DMSO-*d*_6_) δ (ppm): 37.82, 91.02, 104.65, 119.34, 120.04, 122.73, 123.64, 124.51, 126.34, 126.55, 127.13, 129.17, 129.90, 130.05, 130.52, 130.85, 132.25, 135.44, 135.79, 136.25, 142.22, 147.58, 157.39, 161.08, 166.86. Anal. Calcd. For C_34_H_22_FN_5_O_3_S (599.64): C, 68.10; H, 3.70; N, 11.68; S, 5.35. Found: C, 68.30; H, 3.80; N, 11.70; S, 5.45.
*2-((3-Allyl-4-oxo-3,4-dihydroquinazolin-2-yl)thio)-N-(4-(5-cyano-6-oxo-4-phenyl-1,6-dihydropyridin-2-yl)phenyl)acetamide* (**17**). Yellow powder; yield (0.409 g, 75%); m.p.: > 300 °C; ^1^H NMR (500 MHz, DMSO-*d*_6_) δ (ppm): 4.25 (s, 2H, SCH_2_), 4.75 (d, *J* = 5.2 Hz, 2H, CH_2_CH=CH_2_), 5.16–5.27 (m, 2H, CH_2_CH=CH_2_), 5.92–5.98 (m, 1H, CH_2_CH=CH_2_), 6.82 (s, 1H, pyridine-C_5_–H), 7.41–7.48 (m, 4H, Ar-H), 7.57–7.60 (m, 3H, Ar-H), 7.77 (d, *J* = 8.2 Hz, 3H, Ar-H), 7.92 (d, *J* = 8.5 Hz, 2H, Ar-H), 8.08 (d, *J* = 7.9 Hz, 1H, Ar-H), 10.73 (s, 1H, CONH), 12.67 (br s, 1H, pyridine-NH). ^13^C NMR (125 MHz, DMSO-*d*_6_) δ (ppm): 37.20, 46.50, 91.09, 105.51, 114.69, 116.99, 118.17, 118.43, 119.05, 119.63, 125.67, 126.30, 126.79, 127.03, 128.85, 129.14, 130.38, 131.64, 132.49, 135.49, 141.59, 147.16, 156.74, 158.94, 161.01, 161.29, 167.05, 174.30. Anal. Calcd. For C_31_H_23_N_5_O_3_S (545.62): C, 68.24; H, 4.25; N, 12.84; S, 5.88. Found: C, 68.34; H, 4.40; N, 12.90; S, 5.93.
*2-((3-Allyl-4-oxo-3,4-dihydroquinazolin-2-yl)thio)-N-(4-(5-cyano-4-(4-methoxy phenyl)-6-oxo-1,6-dihydropyridin-2-yl)phenyl)acetamide* (**18**). Yellow powder; yield (0.303 g, 80%); m.p.: > 300 °C; ^1^H NMR (500 MHz, DMSO-*d*_6_) δ (ppm): 3.83 (s, 3H, OCH_3_), 4.25 (s, 2H, SCH_2_), 4.73–4.76 (m, 2H, CH_2_CH=CH_2_), 5.15–5.26 (m, 2H, CH_2_CH=CH_2_), 5.91–6.00 (m, 1H, CH_2_CH=CH_2_), 6.71 (s, 1H, pyridine-C_5_–H), 7.05–7.10 (m, 2H, Ar-H), 7.40–7.48 (m, 2H, Ar-H), 7.62–7.67 (m, 2H, Ar-H), 7.71–7.79 (m, 3H, Ar-H), 7.90–7.95 (m, 2H, Ar-H), 8.06–8.10 (m, 1H, Ar-H), 10.70 (s, 1H, CONH), 12.56 (br s, 1H, pyridine-NH). ^13^C NMR (125 MHz, DMSO-*d*_6_) δ (ppm): 37.20, 46.50, 55.90, 91.09, 105.51, 114.69, 118.17, 118.43, 119.05, 119.63, 125.67, 126.30, 126.79, 127.03, 128.85, 129.14, 130.38, 131.64, 132.49, 135.49, 141.59, 147.16, 156.74, 158.94, 161.01, 161.29, 167.05, 174.30. Anal. Calcd. For C_32_H_25_N_5_O_4_S (575.64): C, 66.77; H, 4.38; N, 12.17; S, 5.57. Found: C, 66.80; H, 4.58; N, 12.29; S, 5.70.
*2-((3-Allyl-4-oxo-3,4-dihydroquinazolin-2-yl)thio)-N-(4-(4-(4-chlorophenyl)-5-cyano-6-oxo-1,6-dihydropyridin-2-yl)phenyl)acetamide* (**19**). Yellow powder; yield (0.293 g, 78%); m.p.: > 300 °C; ^1^H NMR (500 MHz, DMSO-*d*_6_) δ (ppm): 4.26 (s, 2H, SCH_2_), 4.74–4.80 (m, 2H, CH_2_CH=CH_2_), 5.16–5.29 (m, 2H, CH_2_CH=CH_2_), 5.91–6.02 (m, 1H, CH_2_CH=CH_2_), 6.83 (s, 1H, pyridine-C_5_–H), 7.41–7.51 (m, 3H, Ar-H), 7.63–7.67 (m, 2H, Ar-H), 7.75–7.77 (m, 2H, Ar-H), 7.77–7.79 (m, 2H, Ar-H), 7.89–7.96 (m, 2H, Ar-H), 8.05–8.10 (m, 1H, Ar-H), 10.75 (s, 1H, CONH), 12.73 (br s, 1H, pyridine-NH). ^13^C NMR (125 MHz, DMSO-*d*_6_) δ (ppm): 37.20, 46.50, 91.54, 105.51, 114.69, 118.17, 118.43, 119.05, 119.63, 126.30, 126.79, 127.03, 128.85, 129.14, 130.38, 131.64, 132.49, 135.49, 141.59, 147.16, 156.74, 158.94, 161.01, 161.29, 167.05, 170.13, 174.30. Anal. Calcd. For C_31_H_22_ClN_5_O_3_S (580.06): C, 64.19; H, 3.82; N, 12.07; S, 5.53. Found: C, 64.22; H, 3.90; N, 12.17; S, 5.64.
*2-((3-Allyl-4-oxo-3,4-dihydroquinazolin-2-yl)thio)-N-(4-(4-(4-bromophenyl)-4-cyano-6-oxo-1,6-dihydropyridin-2-yl)phenyl)acetamide* (**20**). Yellow powder; yield (0.279 g, 80%); m.p.: > 300 °C; ^1^H NMR (500 MHz, DMSO-*d*_6_) δ (ppm): 4.26 (s, 2H, SCH_2_), 4.74–4.80 (m, 2H, CH_2_CH=CH_2_), 5.16–5.29 (m, 2H, CH_2_CH=CH_2_), 5.91–6.02 (m, 1H, CH_2_CH=CH_2_), 6.83 (s, 1H, pyridine-C_5_–H), 7.41–7.51 (m, 3H, Ar-H), 7.63–7.67 (m, 2H, Ar-H), 7.75–7.77 (m, 2H, Ar-H), 7.77–7.79 (m, 2H, Ar-H), 7.89–7.96 (m, 2H, Ar-H), 8.05–8.10 (m, 1H, Ar-H), 10.75 (s, 1H, CONH), 12.73 (br s, 1H, pyridine-NH). ^13^C NMR (125 MHz, DMSO-*d*_6_) δ (ppm): 37.20, 46.50, 91.54, 105.51, 114.69, 118.17, 118.43, 119.05, 119.63, 126.30, 126.79, 127.03, 128.85, 129.14, 130.38, 131.64, 132.49, 135.49, 141.59, 147.16, 156.74, 158.94, 161.01, 161.29, 167.05, 170.13, 174.30. Anal. Calcd. For C_31_H_22_BrN5O_3_S (624.51): C, 59.62; H, 3.55; N, 11.21; S, 5.13. Found: C, 59.70; H, 3.60; N, 11.41; S, 5.26.
*2-((3-Allyl-4-oxo-3,4-dihydroquinazolin-2-yl)thio)-N-(4-(5-cyano-4-(4-fluorophenyl) -6-oxo-1,6-dihydropyridin-2-yl)phenyl)acetamide* (**21**). Yellow powder; yield (0.309 g, 80%); m.p.: > 300 °C; ^1^H NMR (500 MHz, DMSO-*d*_6_) δ (ppm): 4.26 (s, 2H, SCH_2_), 4.74–4.80 (m, 2H, CH_2_CH=CH_2_), 5.16–5.29 (m, 2H, CH_2_CH=CH_2_), 5.91–6.02 (m, 1H, CH_2_CH=CH_2_), 6.83 (s, 1H, pyridine-C_5_–H), 7.41–7.51 (m, 3H, Ar-H), 7.63–7.67 (m, 2H, Ar-H), 7.75–7.77 (m, 2H, Ar-H), 7.77–7.79 (m, 2H, Ar-H), 7.89–7.96 (m, 2H, Ar-H), 8.05–8.10 (m, 1H, Ar-H), 10.75 (s, 1H, CONH), 12.73 (br s, 1H, pyridine-NH). ^13^C NMR (125 MHz, DMSO-*d*_6_) δ (ppm): 37.20, 46.50, 91.54, 105.51, 114.69, 118.17, 118.43, 119.05, 119.63, 126.30, 126.79, 127.03, 128.85, 129.14, 130.38, 131.64, 132.49, 135.49, 141.59, 147.16, 156.74, 158.94, 161.01, 161.29, 167.05, 170.13, 174.30. Anal. Calcd. For C_31_H_22_FN_5_O_3_S (563.61): C, 66.06; H, 3.93; N, 12.43; S, 5.69. Found: C, 66.20; H, 3.95; N, 12.46; S, 5.69.

### 3.2. Biology

#### 3.2.1. Cell Viability Assay

The cell viability test utilized the human mammary gland epithelial cell line (MCF-10A) [62]. Compounds **7**–**21** were incubated with MCF-10A cells for a period of 4 days, followed by an assessment of cell survival through the 3-(4,5-dimethylthiazol-2-yl)-2,5-diphenyltetrazolium bromide (MTT) assay [9]. For more details, see Section A (Supplementary File).

#### 3.2.2. Antiproliferative Assay

Compounds **7**–**21** were examined to determine their ability to inhibit cell proliferation in four types of human cancer cells: pancreatic cancer (Panc-1), breast cancer (MCF-7), colon cancer (HT-29), and epithelial cancer (A-549). The MTT assay [63,64] was employed for this assessment, with Doxorubicin as the reference drug. See Section A (Supplementary File).

#### 3.2.3. EGFR Inhibitory Activity

The most effective antiproliferative hybrids, **8**, **9**, **18**, and **19**, were tested for EGFR-TK inhibitory activity as a possible target for their antiproliferative activity. See Section A (Supplementary File).

#### 3.2.4. BRAF^V600E^ Inhibitory Activity

An in vitro study was carried out to evaluate the activity of the newly synthesized compounds **8**, **9**, **18**, and **19** against BRAF^V600E^ [59]. See Section A (Supplementary File).

### 3.3. In Silico Studies

#### 3.3.1. Docking Study

For the molecular docking study, we used BIOVIA Discovery Studio 2021 software v21.1.0.20.298. The selected proteins underwent preparation for docking analysis through the Protein Preparation Wizard. Subsequently, ligands were mapped onto a three-dimensional model and subjected to energy minimization using LigPrep. To potentially improve binding, a receptor grid was created for the selected binding site using the Receptor Grid Generation Tool. Finally, the Glide tool was employed to evaluate both docking scores and various binding modes for the ligands.

#### 3.3.2. In Silico ADMET Analysis

BIOVIA I Discovery Studio 2016 was used for ADMET investigations. The chemical structures of all substances were imported, and ADMET descriptors were predicted using integrated models that included Lipinski’s Rule of Five and assessments of absorption, distribution, metabolism, excretion, and toxicity. The obtained results were examined to determine the drug-likeness and safety profiles of the compounds.

## 4. Conclusions

A novel series of hybrids, combining quinazolin-4-one and 3-cyanopyrid-2-one in a single compact structure, was synthesized and developed as dual-targeting antiproliferative agents. Using the MTT assay, these newly synthesized compounds were tested for antiproliferative activities against four cancer cell lines. Compounds **18** and **19** were the most potent antiproliferative agents, with GI_50_ values of 1.20 µM and 1.40 µM, respectively, compared to Doxorubicin’s GI_50_ of 1.10 µM. Compounds **18** and **19** suppressed EGFR efficiently, with IC_50_ values of 110 nM and 140 nM, respectively. Furthermore, with IC_50_ values of 140 nM and 190 nM, these hybrid compounds showed promising inhibition of BRAF^V600E^. As a result of their combined inhibition of EGFR and BRAF^V600E^, they have the potential to operate as effective antiproliferative agents. Molecular docking studies indicated that compound **18** is a potent inhibitor for EGFR and BRAF^V600E^ kinase domains. Moreover, an ADMET study suggested that most compounds have low toxicity and limited adverse effects in the tested models. Additional in vitro and in vivo studies and potential chemical modifications may be required to advance the development of highly effective antiproliferative medicines.

## Figures and Tables

**Table 1:** Cytotoxicity assay of compounds **7**–**21** and Doxorubicin in non-tumorigenic MCF-10A cells.

Compound	Cell Viability %
**7**	90
**8**	89
**9**	96
**10**	88
**11**	91
**12**	86
**13**	87
**14**	89
**15**	87
**16**	89
**17**	89
**18**	90
**19**	91
**20**	91
**21**	87
**Doxorubicin**	^a^ ND

^a^ ND: Not determined.

**Table 2:** Antiproliferative activity of compounds **7**–**21** and Doxorubicin.

Comp.	Antiproliferative Activity ^a^ IC_50_ ± SEM (µM)				
	^b^ A-549	^c^ MCF-7	^d^ Panc-1	^e^ HT-29	^f^ Average IC_50_(GI_50_)
**7**	9.20 ± 0.80	9.10 ± 0.80	9.30 ± 0.80	9.80 ± 0.90	9.30
**8**	1.50 ± 0.10	1.40 ± 0.10	1.70 ± 0.10	1.70 ± 0.10	1.60
**9**	1.80 ± 0.10	1.60 ± 0.10	2.00 ± 0.10	2.00 ± 0.10	1.80
**10**	2.40 ± 0.20	2.20 ± 0.45	2.60 ± 0.20	2.70 ± 0.20	2.50
**11**	3.70 ± 0.30	3.60 ± 0.30	3.80 ± 0.30	4.00 ± 0.30	3.80
**12**	7.50 ± 0.60	7.20 ± 0.70	7.80 ± 0.70	7.70 ± 0.70	7.50
**13**	3.20 ± 0.30	3.10 ± 0.30	3.40 ± 0.30	3.50 ± 0.30	3.30
**14**	4.10 ± 0.40	3.90 ± 0.40	4.20 ± 0.40	4.20 ± 0.40	4.10
**15**	4.70 ± 0.40	4.60 ± 0.40	5.00 ± 0.40	4.90 ± 0.40	4.80
**16**	4.80 ± 0.50	4.70 ± 0.40	5.10 ± 0.50	5.10 ± 0.50	4.90
**17**	8.40 ± 0.70	8.10 ± 0.70	8.90 ± 0.70	9.00 ± 0.70	8.60
**18**	1.10 ± 0.10	1.00 ± 0.10	1.30 ± 0.10	1.30 ± 0.10	1.20
**19**	1.30 ± 0.10	1.20 ± 0.08	1.60 ± 0.10	1.60 ± 0.10	1.40
**20**	2.00 ± 0.10	1.80 ± 0.10	2.10 ± 0.10	2.10 ± 0.10	2.00
**21**	2.10 ± 0.20	1.90 ± 0.10	2.20 ± 0.20	2.20 ± 0.10	2.10
**Doxorubicin**	1.20 ± 0.10	0.90± 0.10	1.40 ± 0.10	1.00 ± 0.10	1.10

^a^ IC_50_ (μM): Expressed as mean ± SEM. ^b^ A-549: Human lung carcinoma cell line. ^c^ MCF-7: Human breast adenocarcinoma cell line. ^d^ Panc-1: Human pancreatic cancer cell line. ^e^ HT-29: Human colon cancer cell line. ^f^ GI_50_: average of IC_50_ against four cancer cell line.

**Table 3:** Effects of compounds **8**, **9**, **18**, **19,** and Erlotinib on EGFR and BRAF^V600E^.

Compound	EGFR Inhibition ^a^ IC_5_ ± SEM (nM)	BRAF Inhibition IC_50_ ± SEM (nM)
**8**	160 ± 12	220 ± 15
**9**	190 ± 15	290 ± 20
**18**	110 ± 10	140 ± 10
**19**	140 ± 11	190 ± 12
**Erlotinib**	80 ± 5	60 ± 5

^a^ IC_50_ (nM): Expressed as mean ± SEM.

**Table 4:** Predicted ADMET for synthesized qunazolin-4-one/3-cyanopyrid-2-one hybrids **7**–**21**.

Comp. ID	PSA	PPB ^a^	Absorption Level ^b^	CYP2D6 Prediction ^c^	BBB Level ^d^	Solubility Level ^e^	AlogP98
**7**	113.584	True	2	False	4	2	5.009
**8**	122.514	True	2	False	4	2	4.992
**9**	113.584	True	3	False	4	1	5.673
**10**	113.584	True	2	False	4	1	5.757
**11**	113.584	True	2	False	4	2	5.214
**12**	115.133	False	3	False	4	2	5.361
**13**	124.063	False	3	False	4	2	5.345
**14**	115.133	True	2	False	4	1	6.026
**15**	115.133	False	2	False	4	1	6.11
**16**	115.133	True	2	False	4	2	5.567
**17**	115.133	False	1	False	4	2	4.402
**18**	124.063	False	2	False	4	2	4.385
**19**	115.133	True	2	False	4	2	5.066
**20**	115.133	False	2	False	4	2	5.15
**21**	115.133	True	2	False	4	2	4.607
**Erlotinib**	70.844	True	0	False	1	2	4.299

^a^ PBB, plasma protein binding, FALSE means less than 90%, TRUE means > 90%; ^b^ Absorption level, 0 = good, 1 = moderate, 2 = poor, 3 = very poor; ^c^ CYP2D6, cytochrome P2D6, TRUE = inhibitor, FALSE = non inhibitor; ^d^ BBB level, blood–brain barrier level, 0 = very high, 1 = high, 2 = medium, 3 = low, 4 = very low; ^e^ Solubility level, 1 = very low, 2 = low, 3 = good, 4 = optimal.

**Table 5:** The predicted toxicity properties of the synthesized compounds.

Comp. ID	FDA Rodent Carcinogenicity (Mouse- Female)	Rat Maximum Tolerated Dose (Feed) ^a^	Rat Oral LD_50_^a^	Ocular Irritancy	Skin Irritancy
**7**	Non-Carcinogen	0.0306	0.592702	Mild	Non-Irritant
**8**	Non-Carcinogen	0.0164	0.63625	Mild	Non-Irritant
**9**	Non-Carcinogen	0.0367	0.686439	Mild	Non-Irritant
**10**	Non-Carcinogen	0.0236	0.912908	Mild	Non-Irritant
**11**	Non-Carcinogen	0.0400	0.357337	Mild	Non-Irritant
**12**	Non-Carcinogen	0.0239	1.5471	Mild	Non-Irritant
**13**	Non-Carcinogen	0.0127	0.806376	Mild	Non-Irritant
**14**	Non-Carcinogen	0.0285	0.737159	Mild	Non-Irritant
**15**	Non-Carcinogen	0.0182	0.974688	Mild	Non-Irritant
**16**	Non-Carcinogen	0.0311	0.384654	Mild	Non-Irritant
**17**	Non-Carcinogen	0.0251	0.719247	Mild	Non-Irritant
**18**	Non-Carcinogen	0.0134	0.582941	Mild	Non-Irritant
**19**	Non-Carcinogen	0.0300	0.533142	Moderate-Severe	Non-Irritant
**20**	Non-Carcinogen	0.0192	0.707879	Moderate-Severe	Non-Irritant
**21**	Non-Carcinogen	0.0327	0.277722	Moderate-Severe	Non-Irritant
**Erlotinib**	Non-Carcinogen	0.0730	0.534347	Mild	Non-Irritant

^a^ Unit: g/kg body weight.

## Data Availability

Data is contained within the article and supplementary material.

## Supplementary Materials

The following supporting information can be downloaded at: https://www.mdpi.com/article/10.3390/ph16111522/s1.
