# Supplementary material for: Quinazolin-4-one/3-cyanopyridin-2-one Hybrids as Dual Inhibitors of EGFR and BRAFV600E: Design, Synthesis, and Antiproliferative Activity
Source: Pharmaceuticals (Basel). 2023 Oct 26;16(11):1522. doi: 10.3390/ph16111522 (PMC10674657; doi:10.3390/ph16111522)
Supplement: Supplementary file 1 [file pharmaceuticals-16-01522-s001.zip › pharmaceuticals-2645771-supplementary.pdf]

Quinazolin-4-one/3-cyanopyridin-2-one Hybrids as Dual Inhibitors of EGFR and BRAF^{V600E}: Design, Synthesis, and Antiproliferative Activity

Compound 7

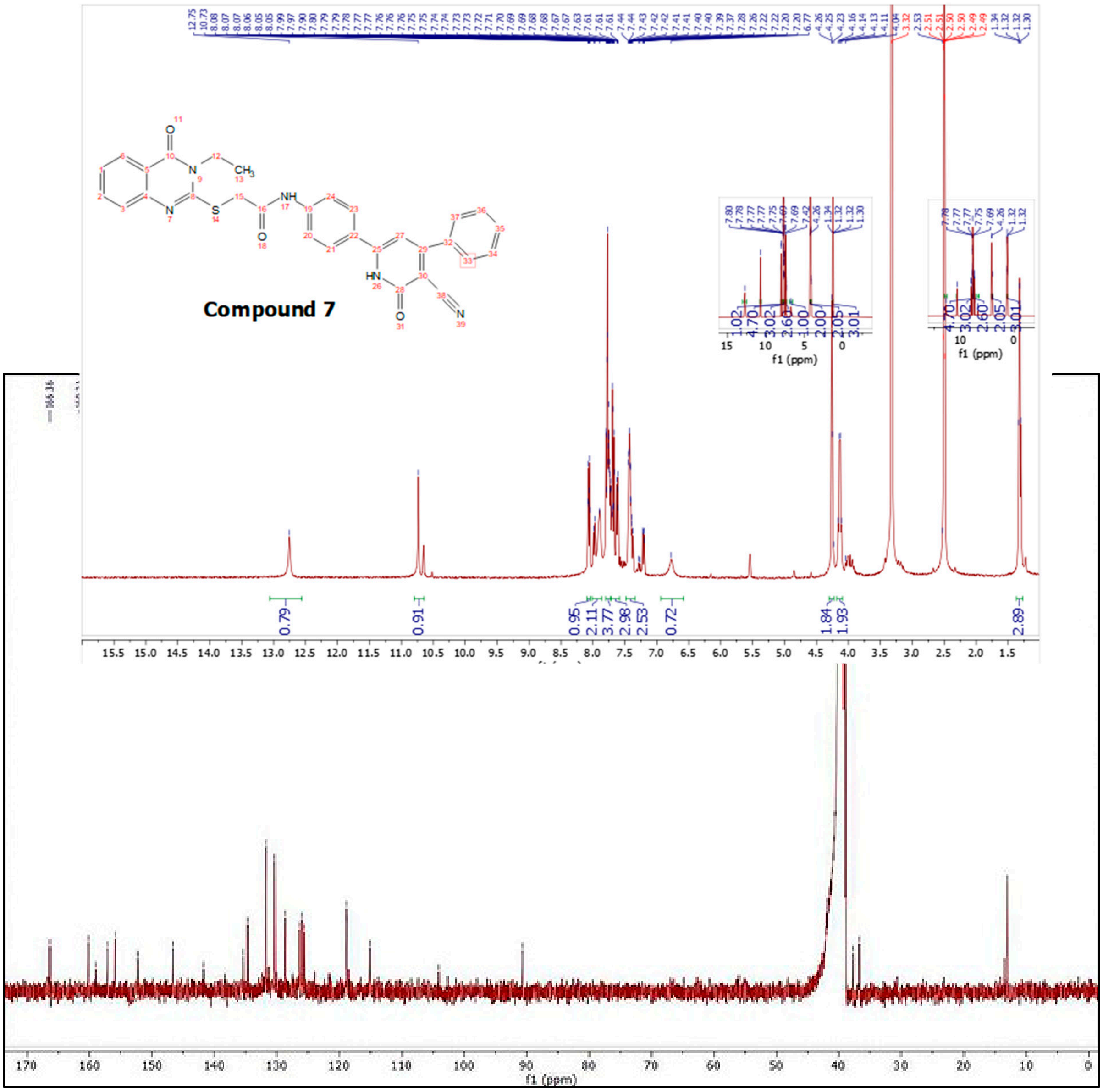

Compound 8

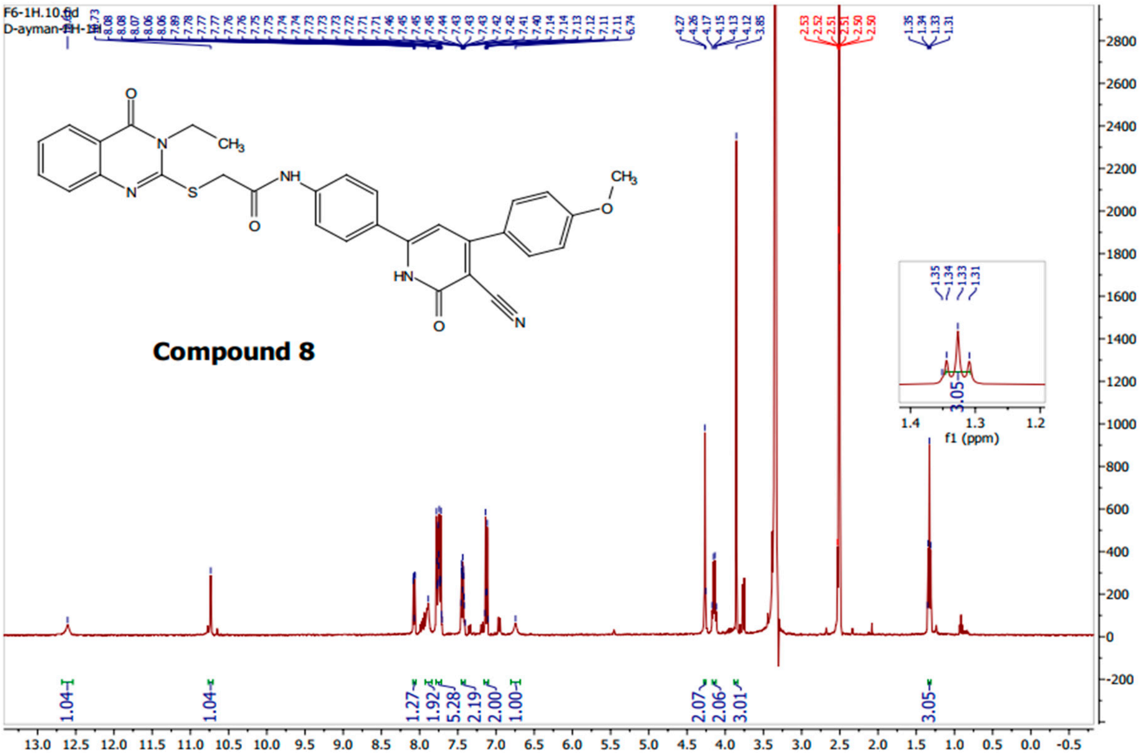

F6-1H.10.fid
Compound 8
1.04
1.04
1.27
1.92
5.28
2.19
2.00
1.00
2.07
2.06
3.01
3.05
f1 (ppm)

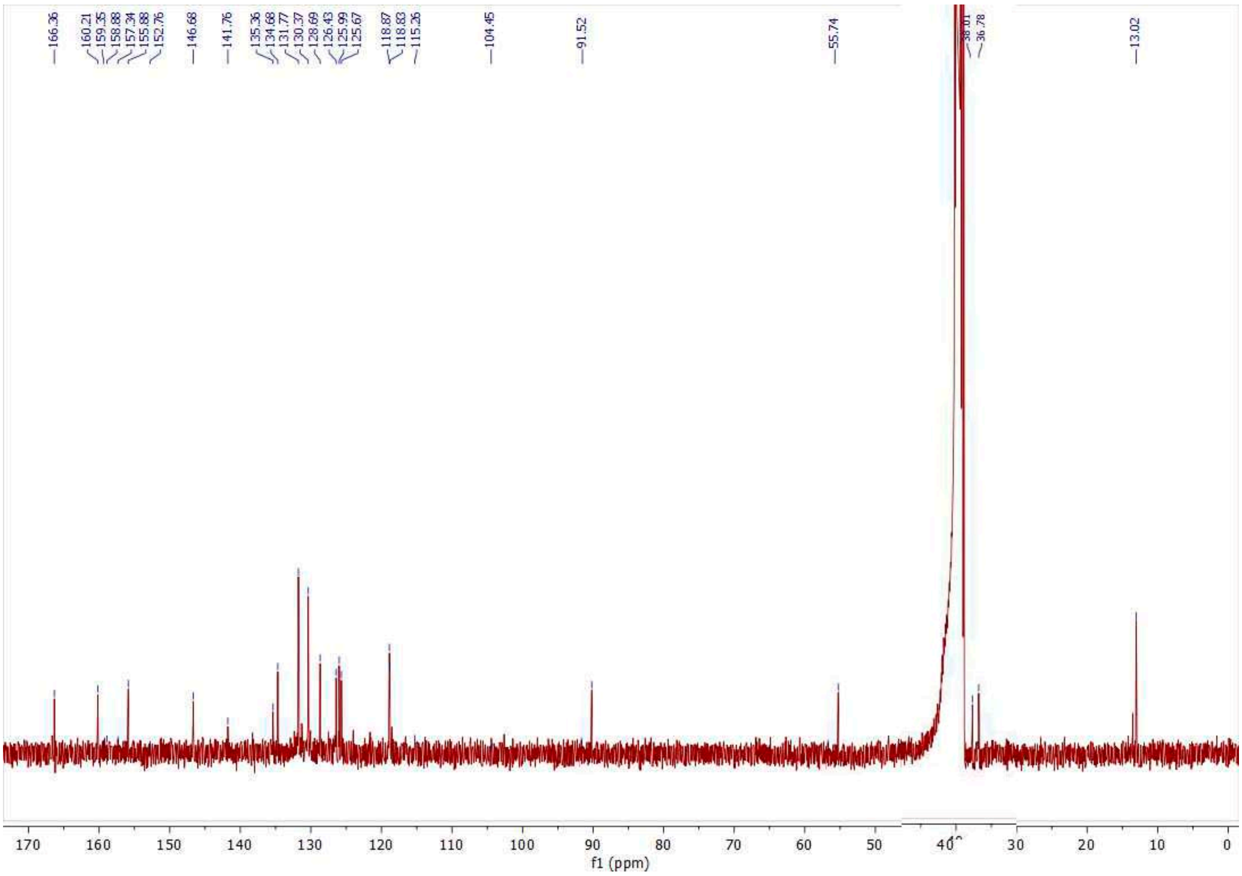

166.36
160.21
159.35
158.88
157.34
155.88
152.76
146.68
141.76
135.36
134.68
131.77
130.37
128.69
126.43
125.99
125.67
118.87
118.83
115.26
104.45
91.52
55.74
36.78
13.02
f1 (ppm)

Compound 9

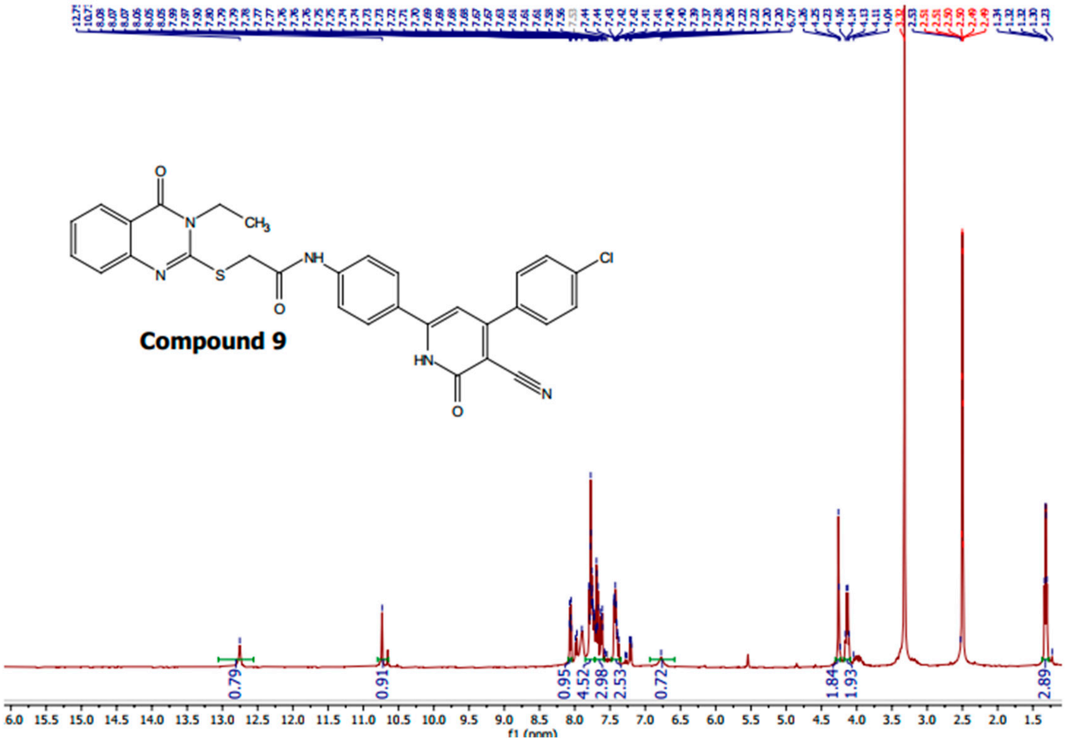

Compound 9
NH
HN
CH3
Cl
0.79
0.91
0.95
4.52
2.98
2.53
0.72
1.84
1.93
2.89
f1 (ppm)

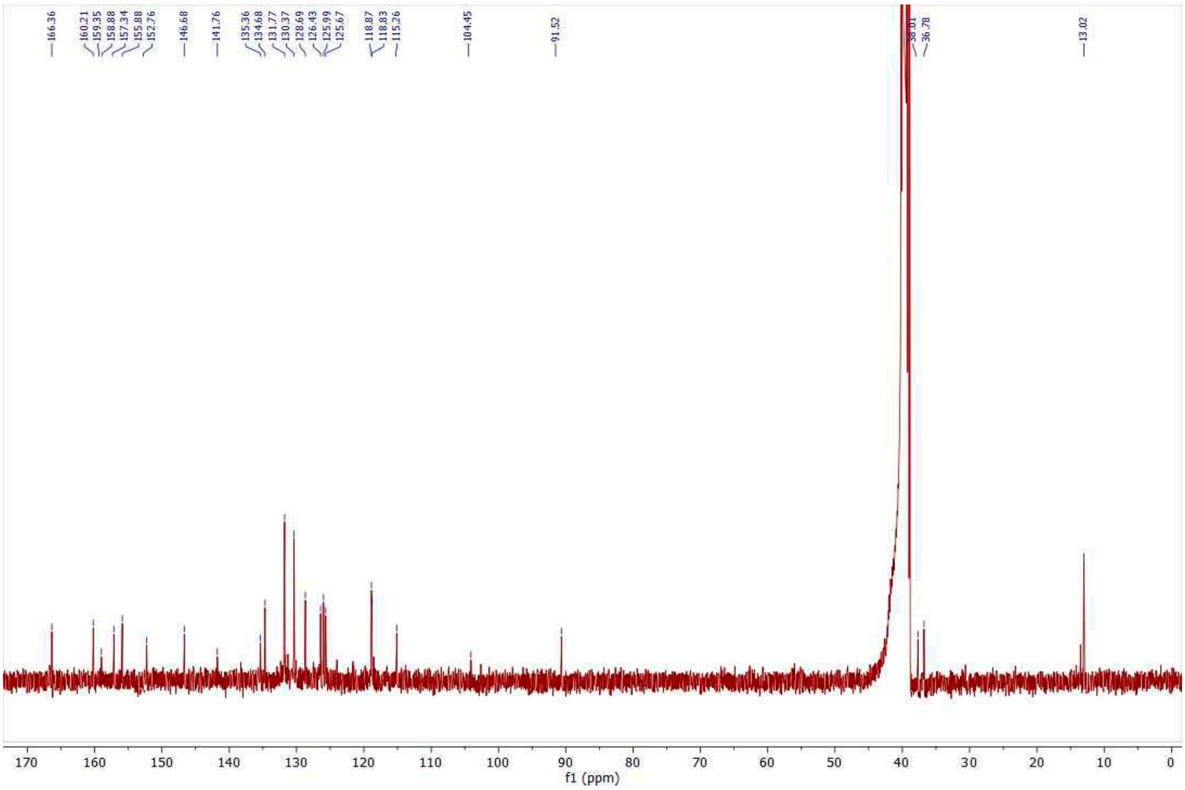

f1 (ppm)

Compound 10

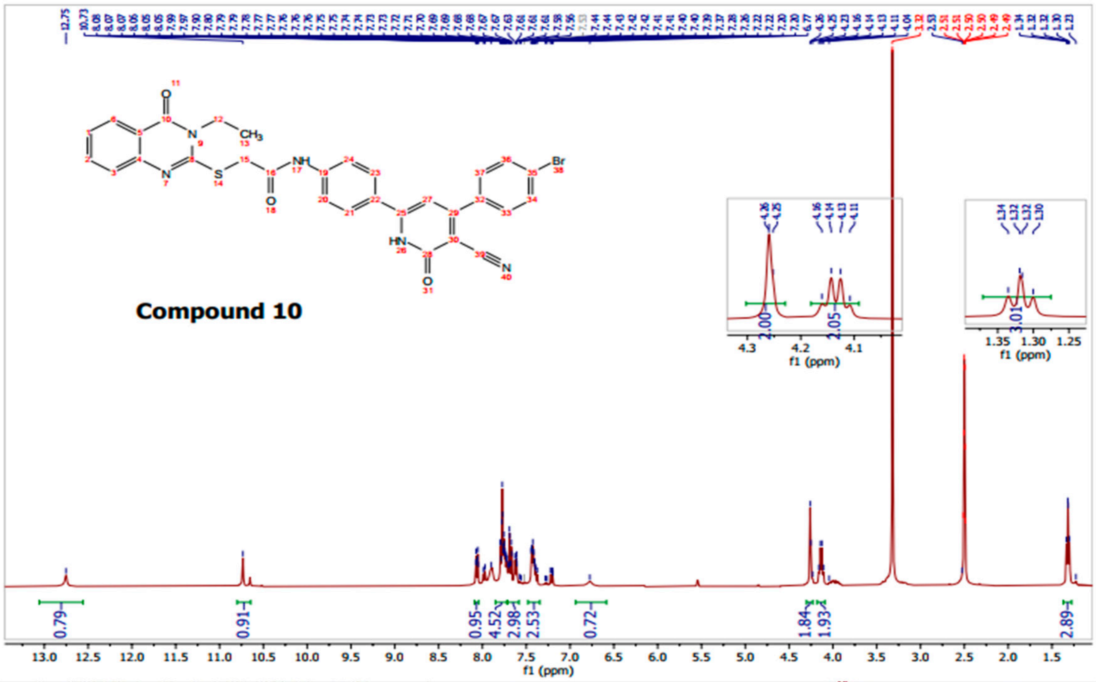

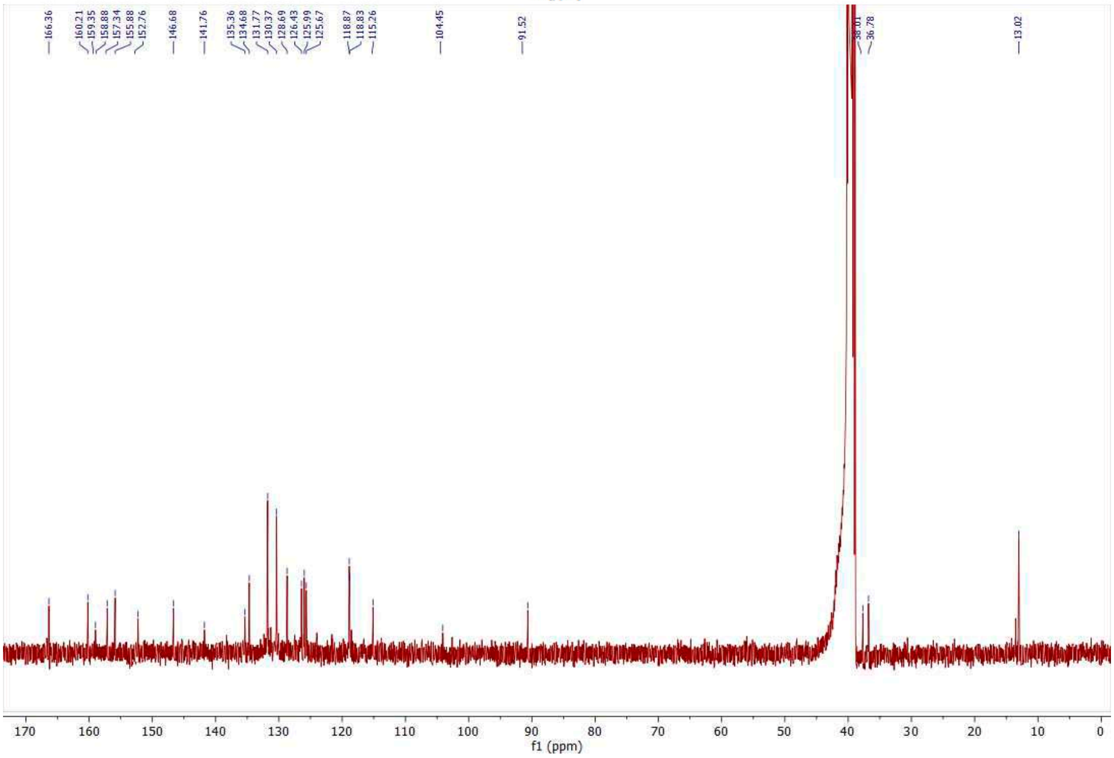

Compound 11

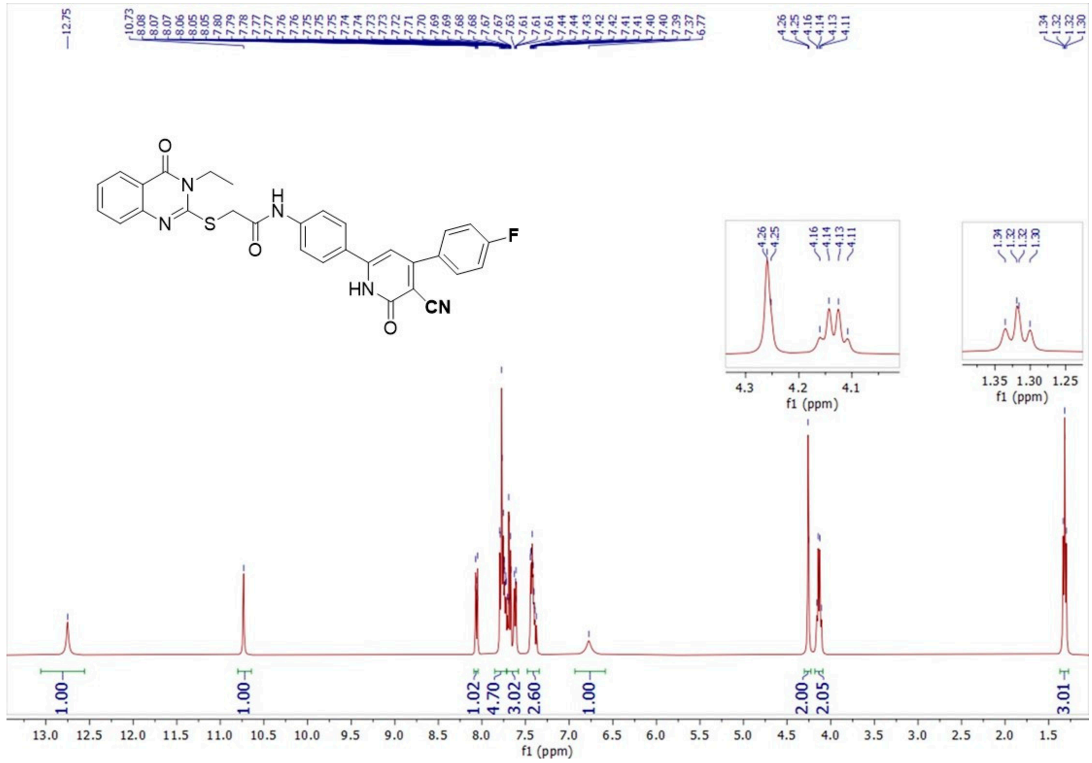

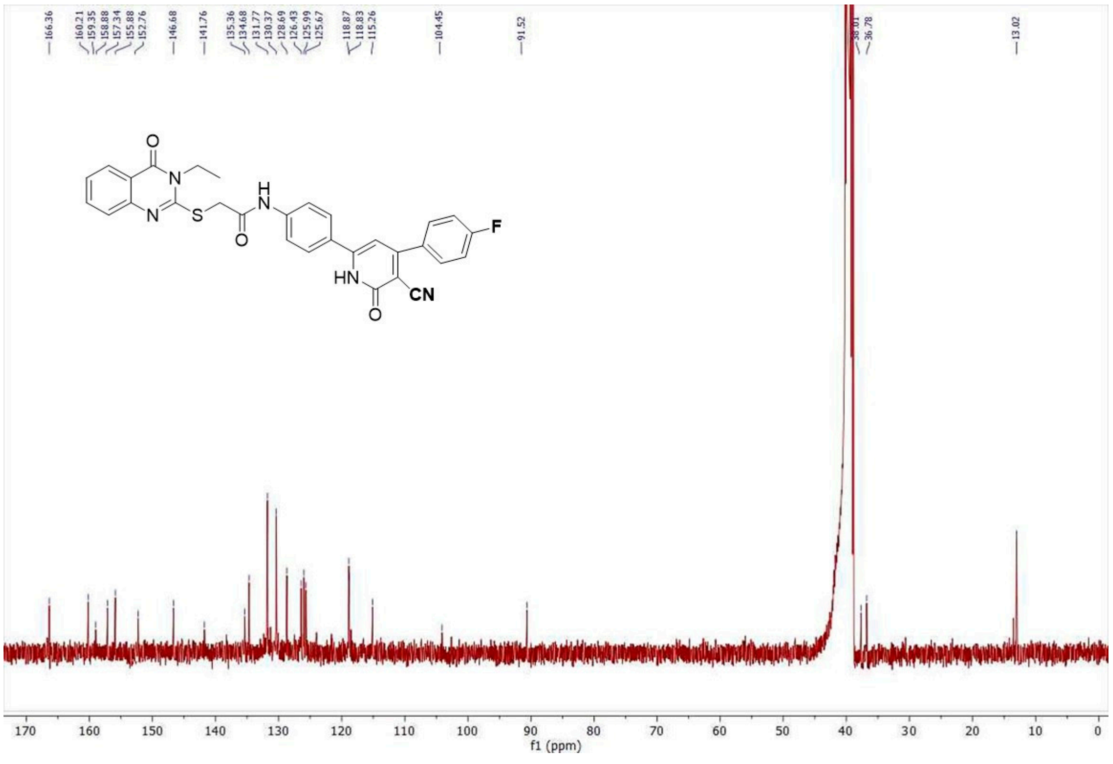

Compound 12

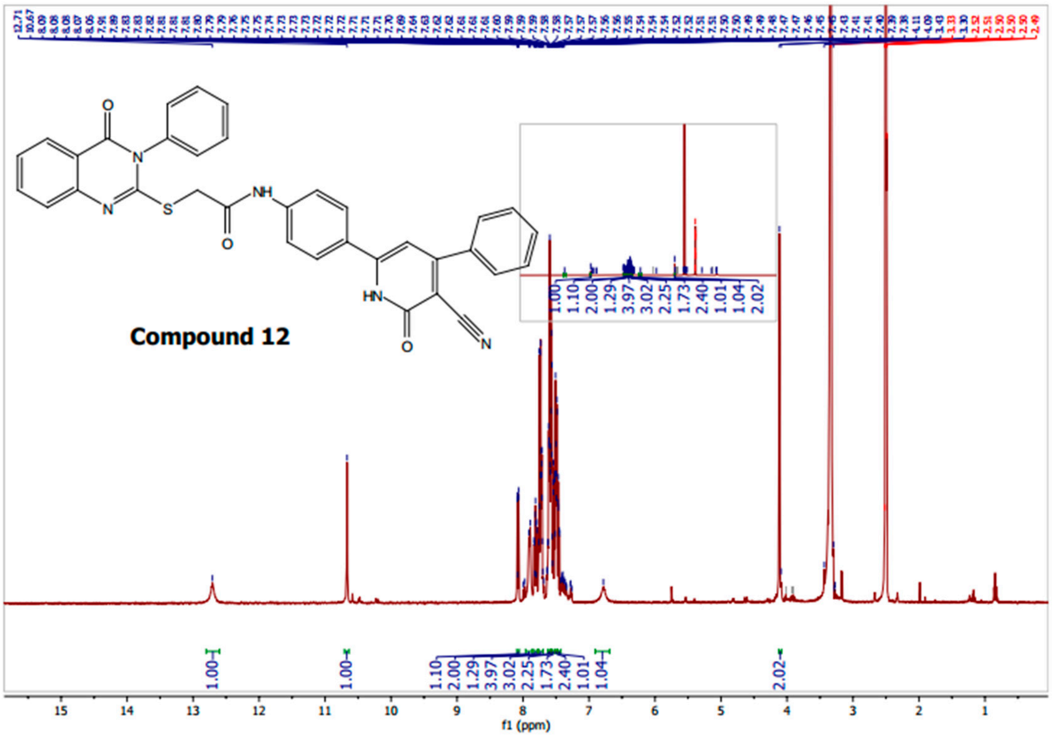
Compound 12
O
N
N
S
NH
O
HN
O
N
1.00
1.10
2.00
1.29
3.97
3.02
2.25
1.73
2.40
1.01
1.04
2.02
1.00
1.00
15 14 13 12 11 10 9 8 7 6 5 4 3 2 1
f1 (ppm)

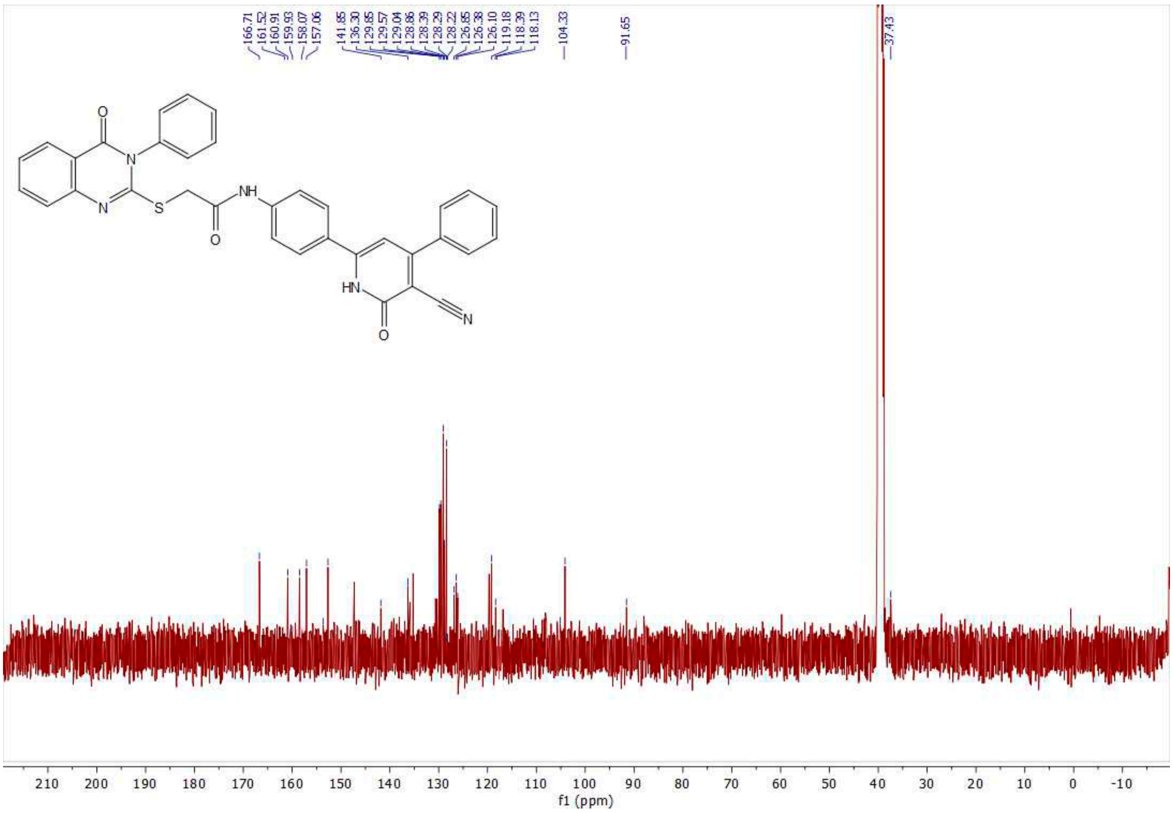
166.71
161.52
160.91
159.93
158.07
157.06
141.85
136.30
129.85
129.57
129.04
128.86
128.39
128.29
128.22
126.65
126.38
126.10
119.18
118.39
118.13
104.33
91.65
37.43
O
N
N
S
NH
O
HN
O
N
210 200 190 180 170 160 150 140 130 120 110 100 90 80 70 60 50 40 30 20 10 0 -10
f1 (ppm)

Compound 13

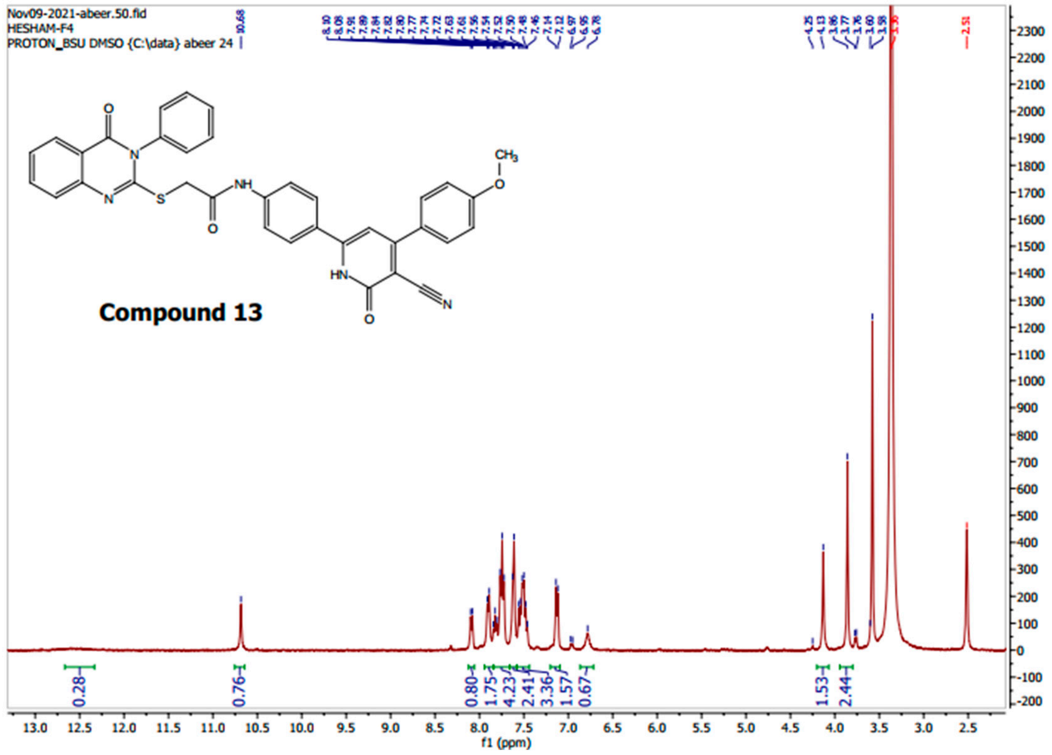
Nov09-2021-abeer.50.fid
HESHAM-F4
PROTON_BSU DMSO {C:\data} abeer 24
Compound 13
f1 (ppm)

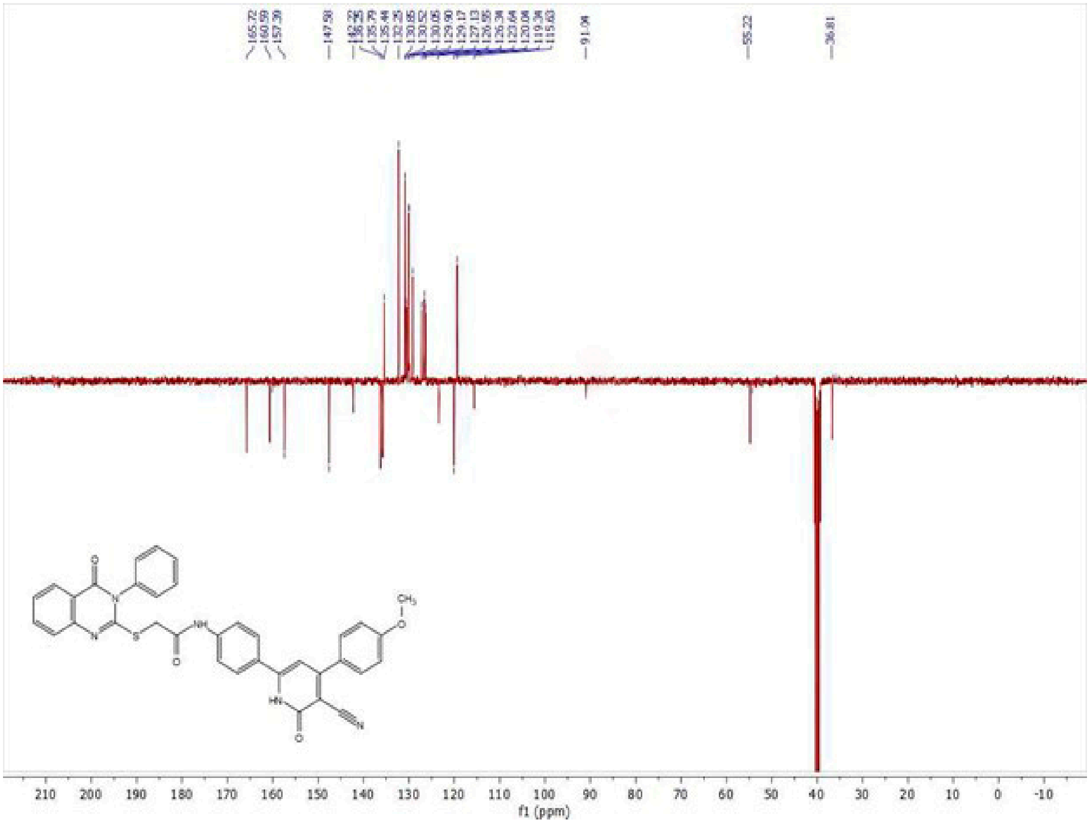
f1 (ppm)

Compound 14

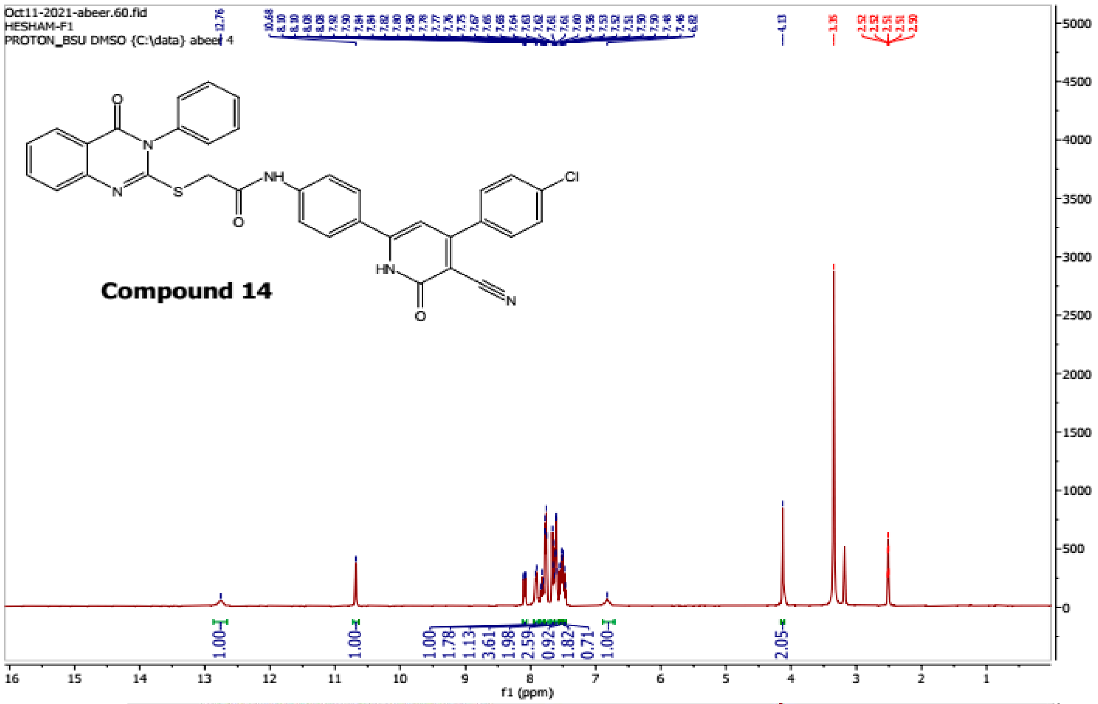
Oct11-2021-abeer.60.fid
HESHAM-F1
PROTON_BSU DMSO {C:\data} abeer 4
Compound 14
f1 (ppm)

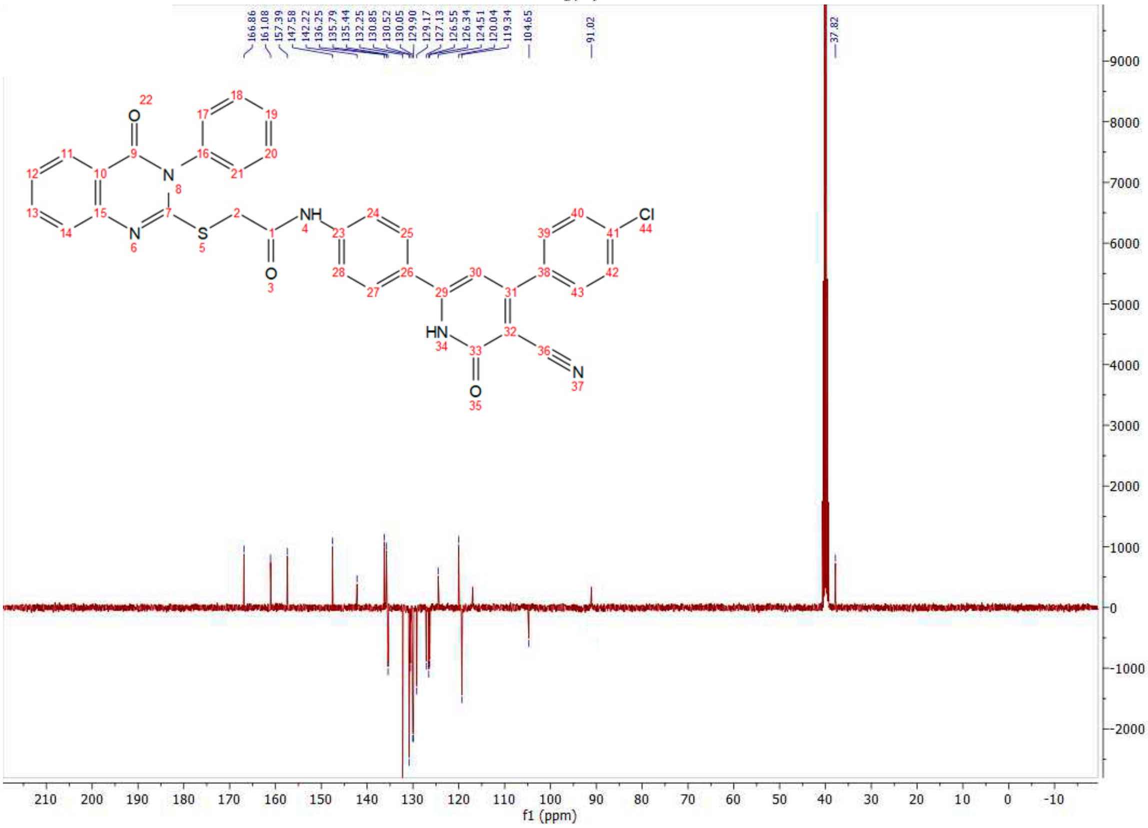
f1 (ppm)

Compound 15

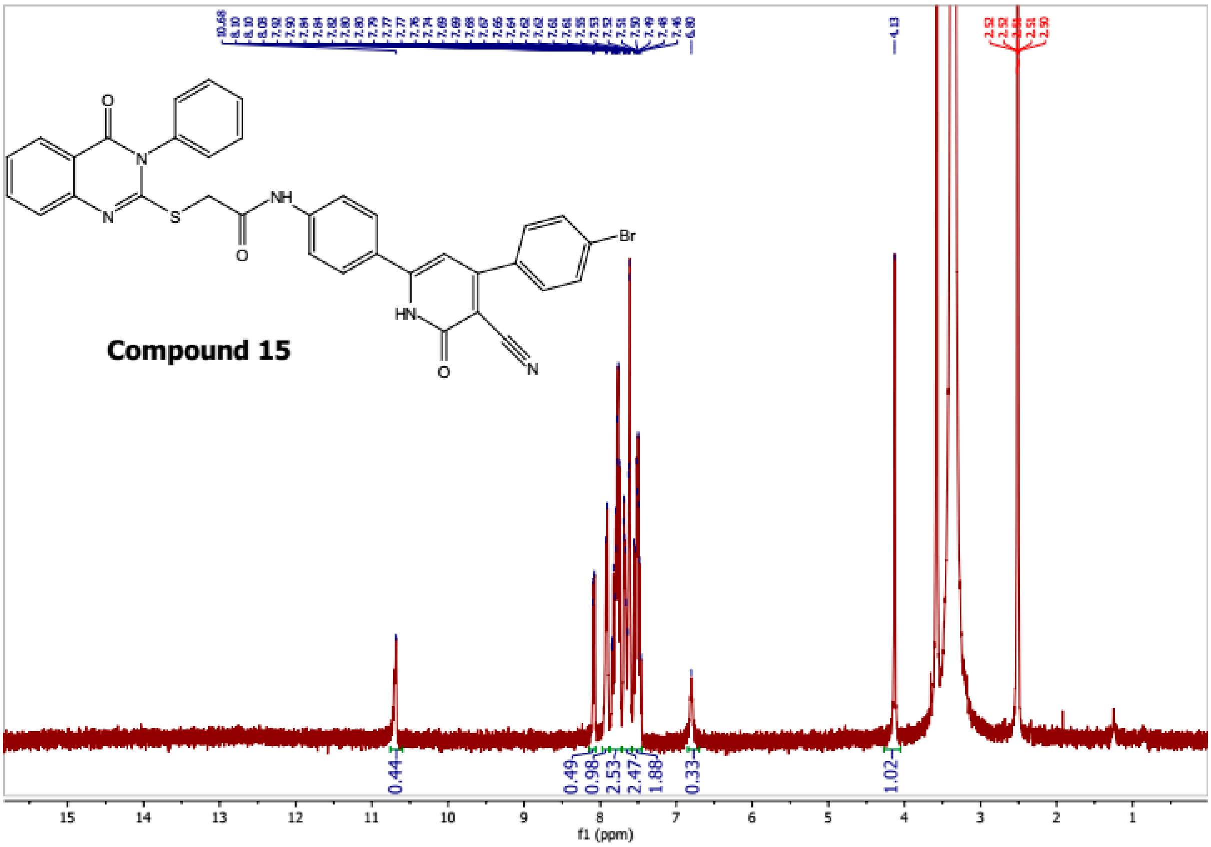

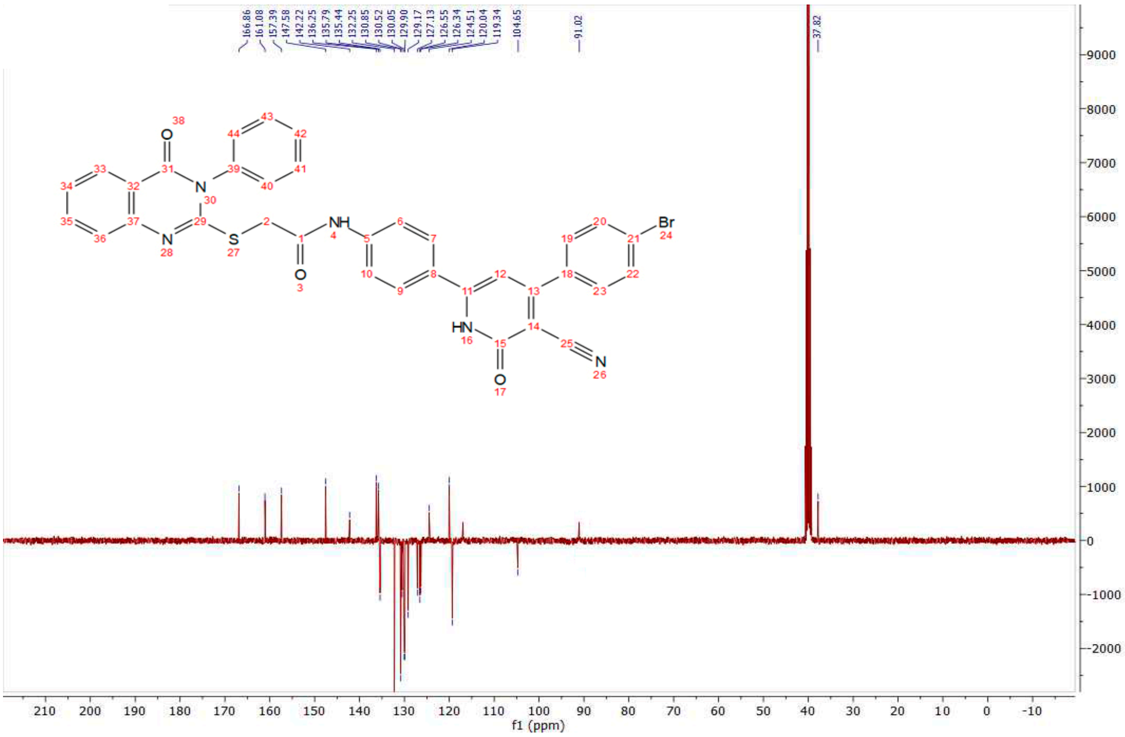

Compound 16

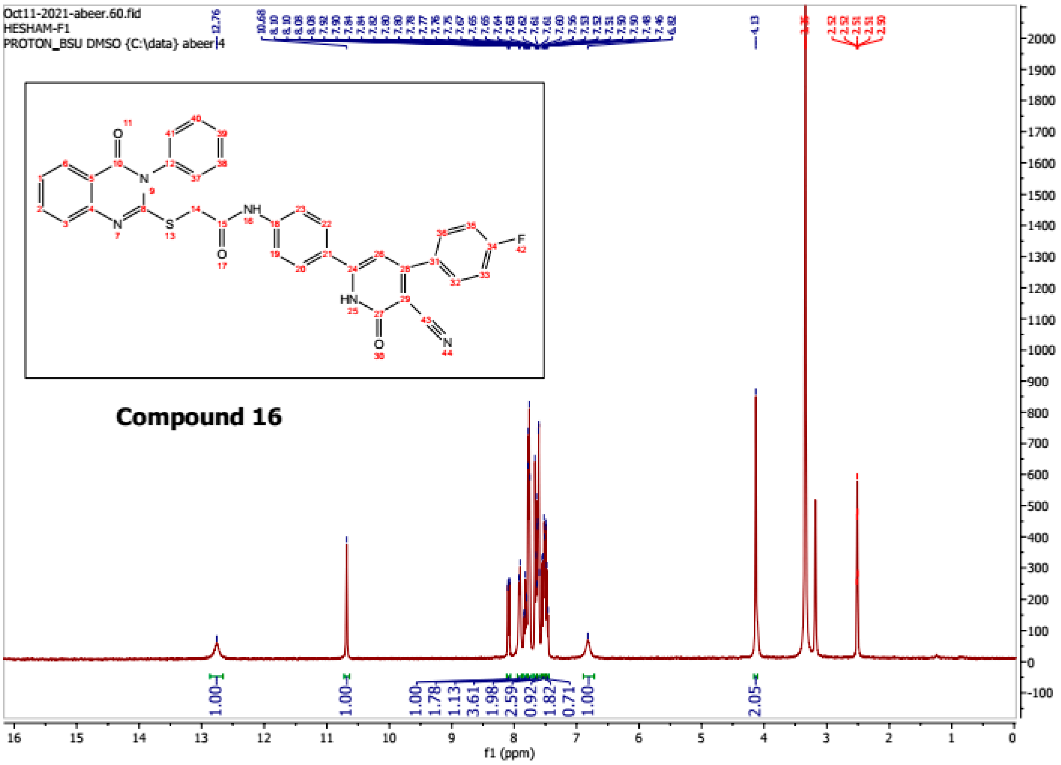

Oct11-2021-abeer.60.fid
HESHAM-F1
PROTON_BSU DMSO {C:\data} abeer 4
Compound 16
f1 (ppm)

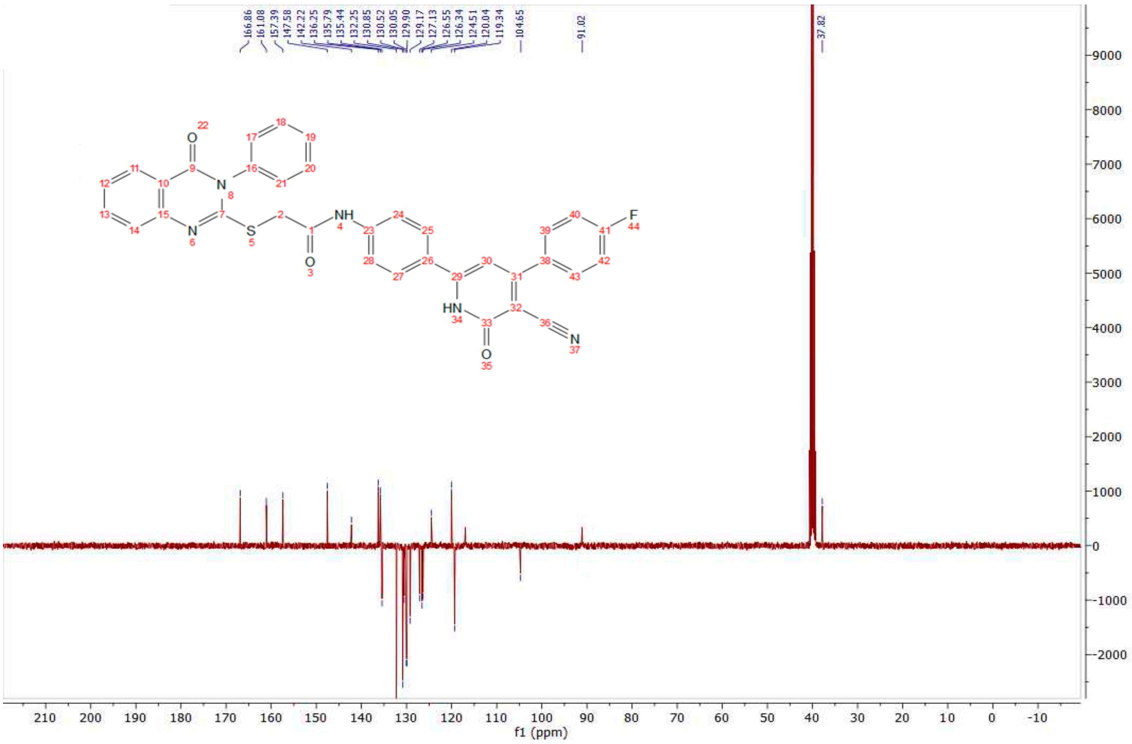

f1 (ppm)

Compound 17

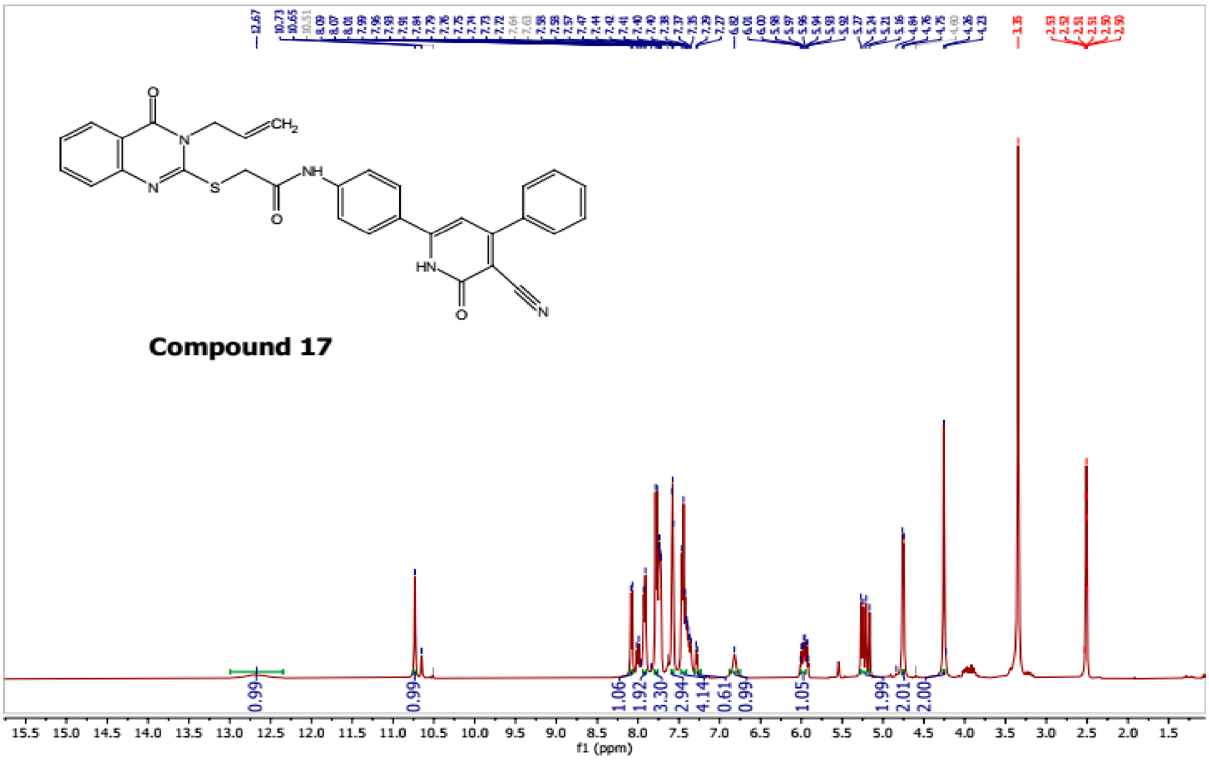

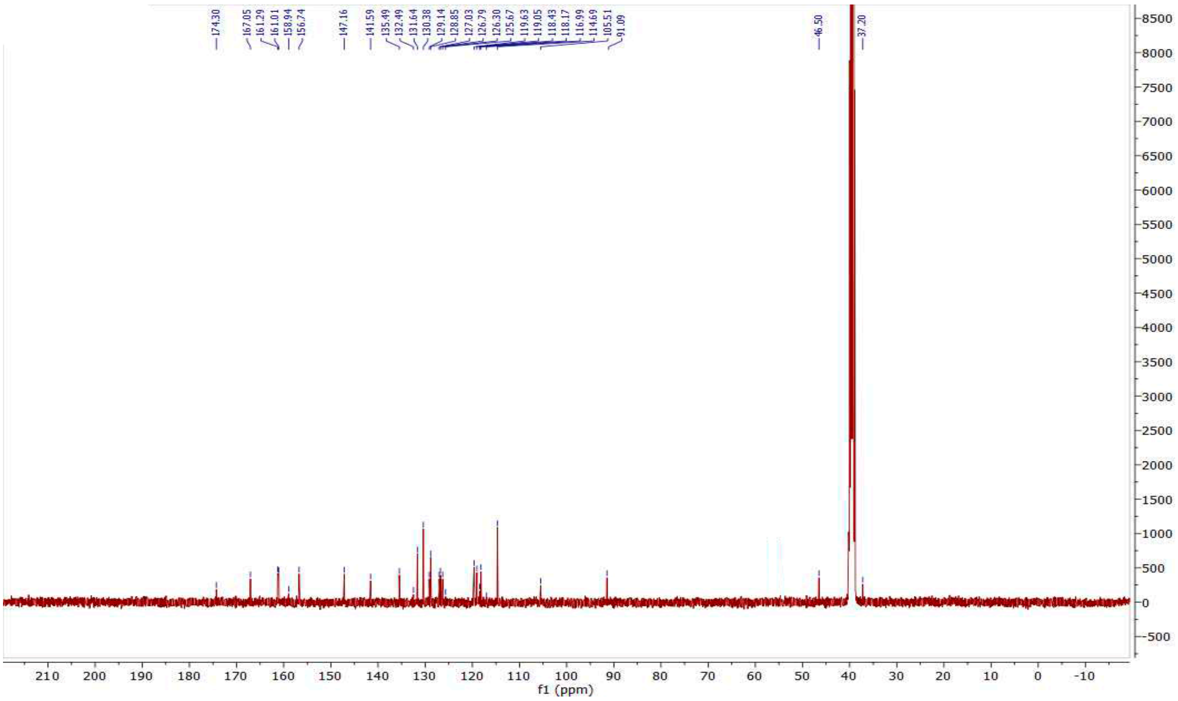

Compound 18

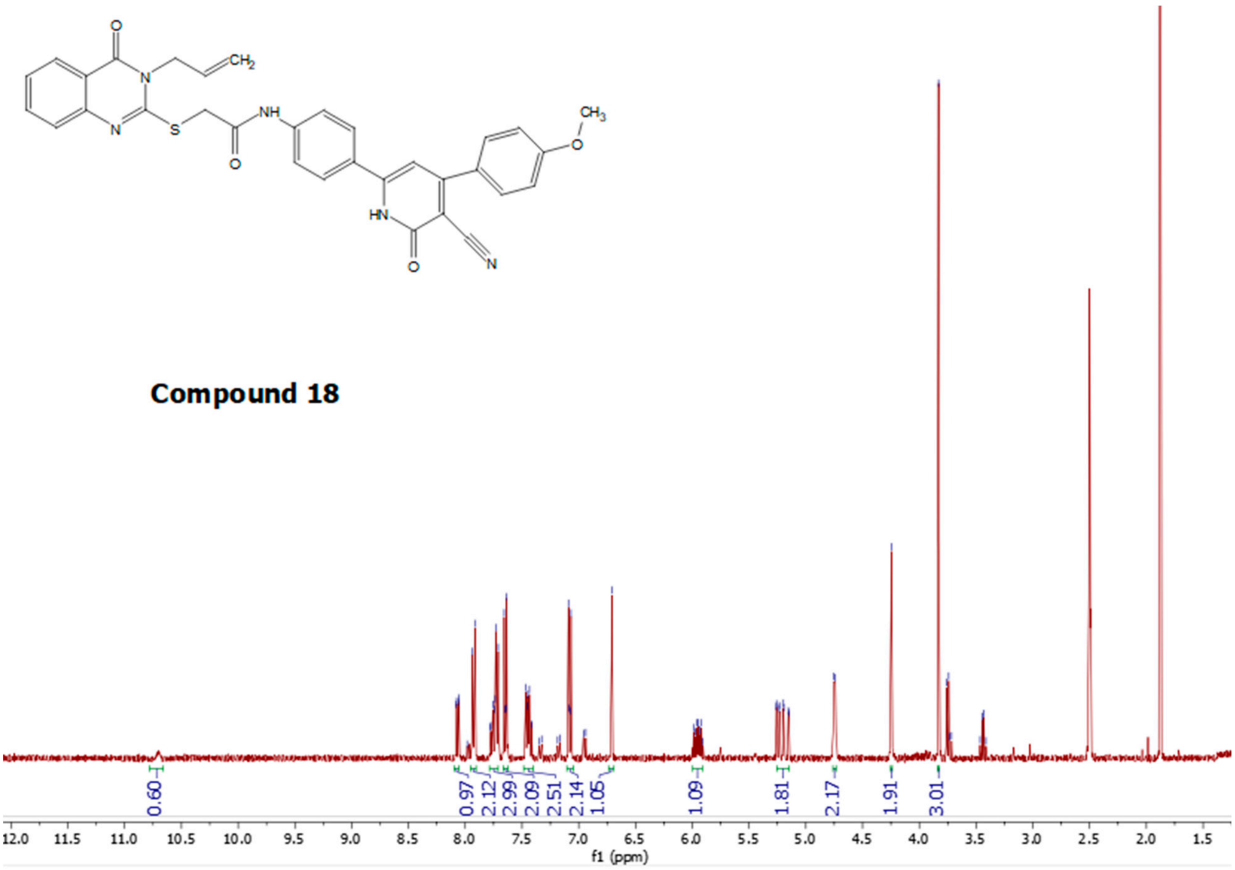

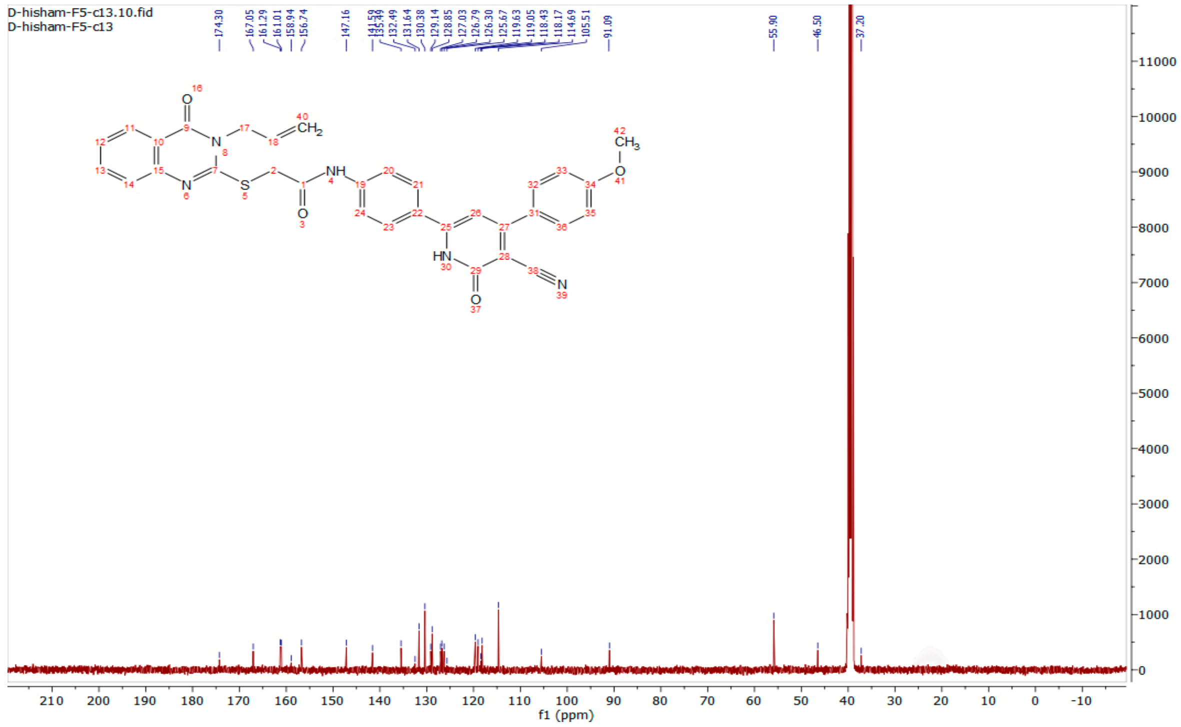

Compound 19

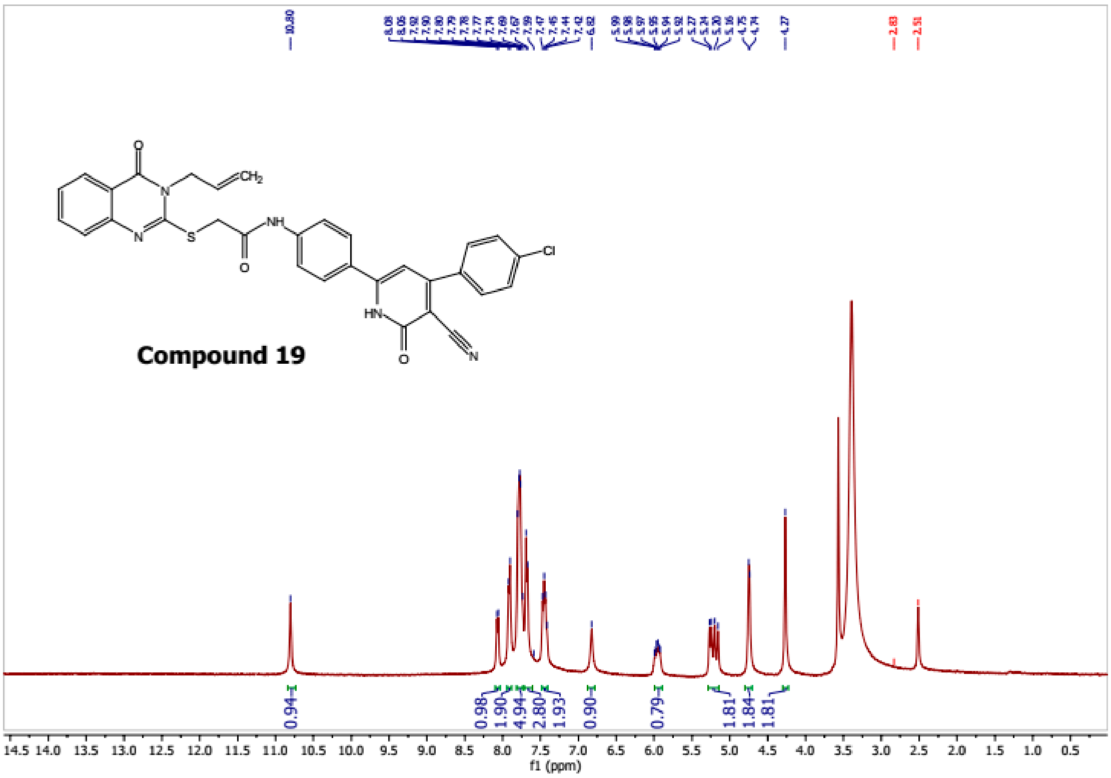

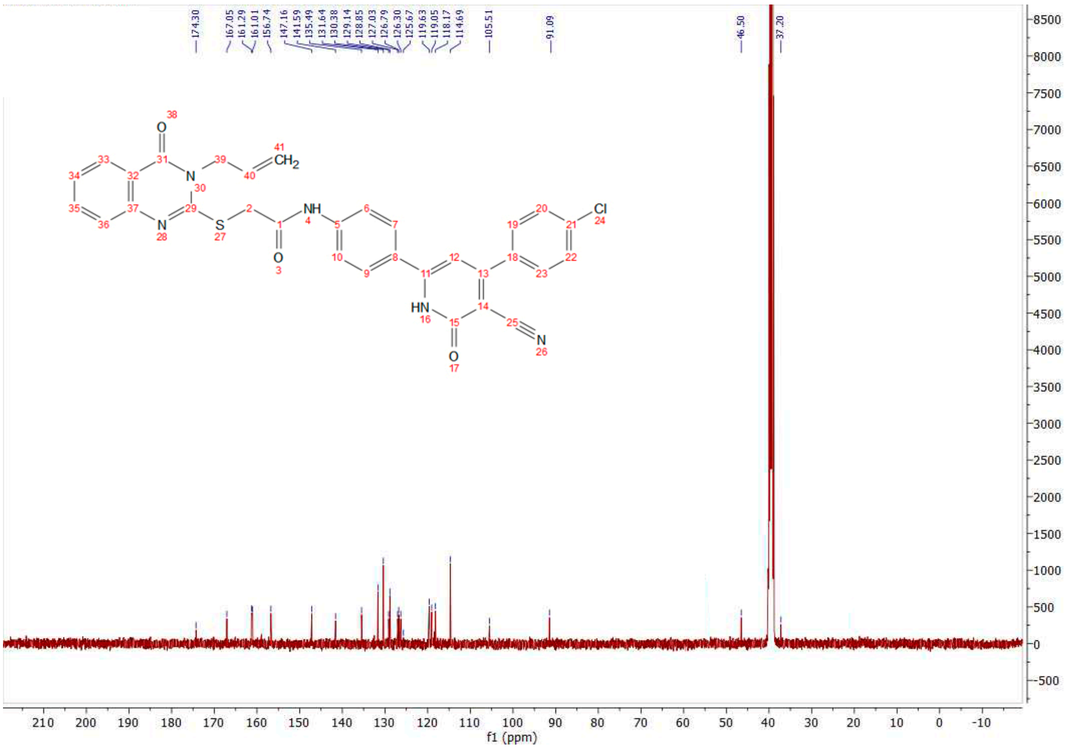

Compound 20

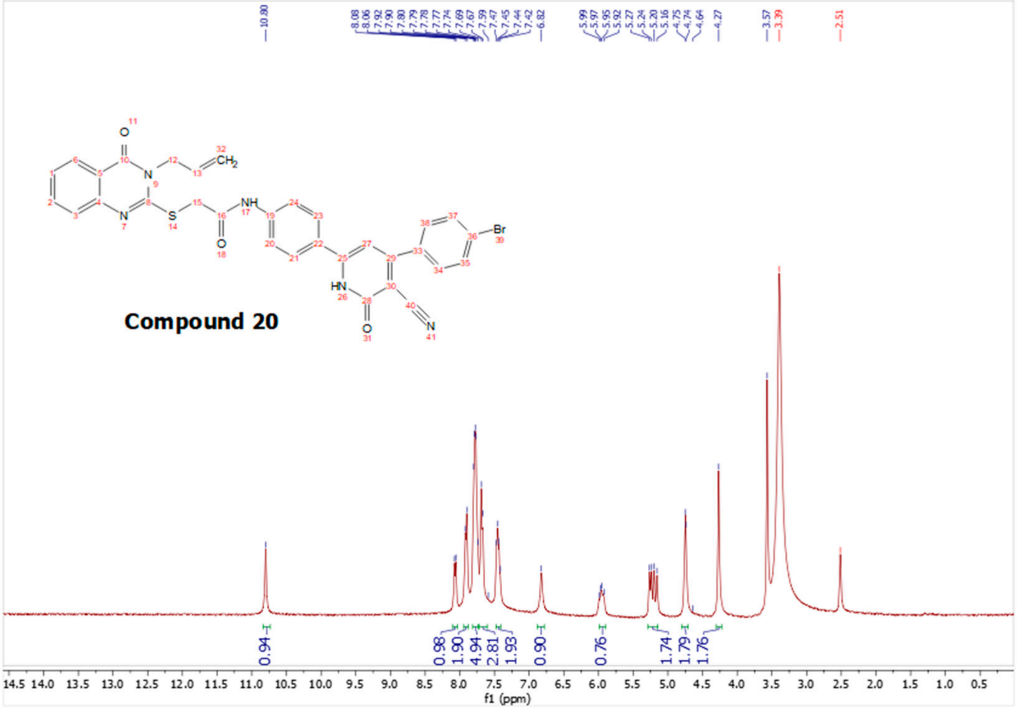

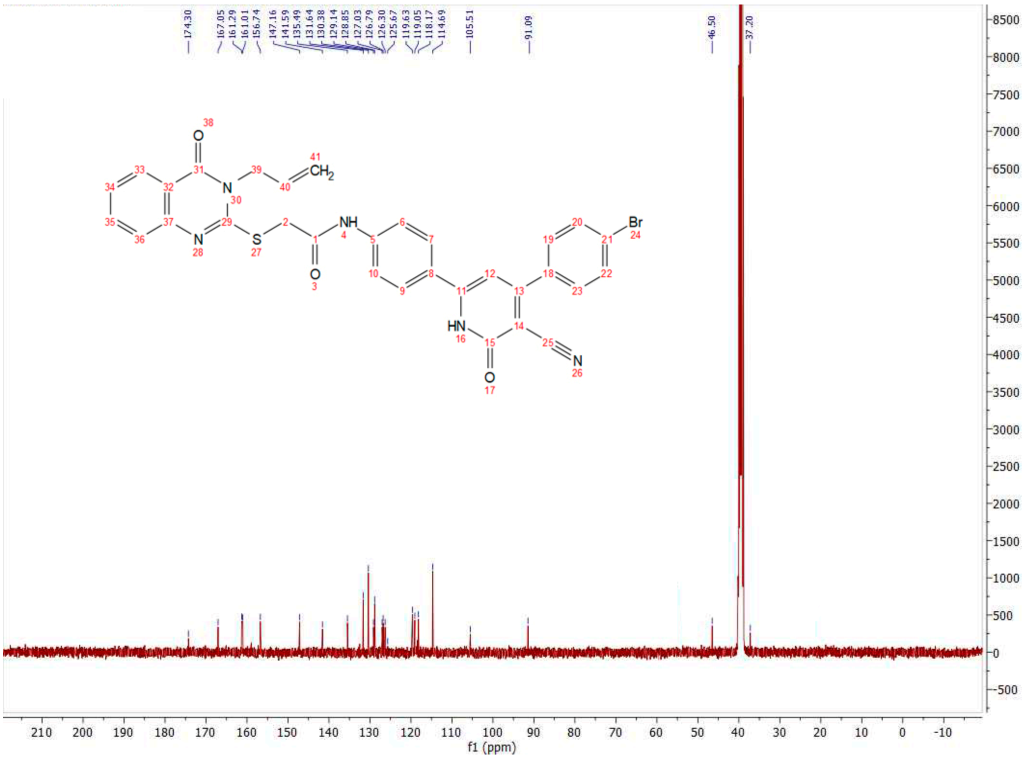

Compound 21

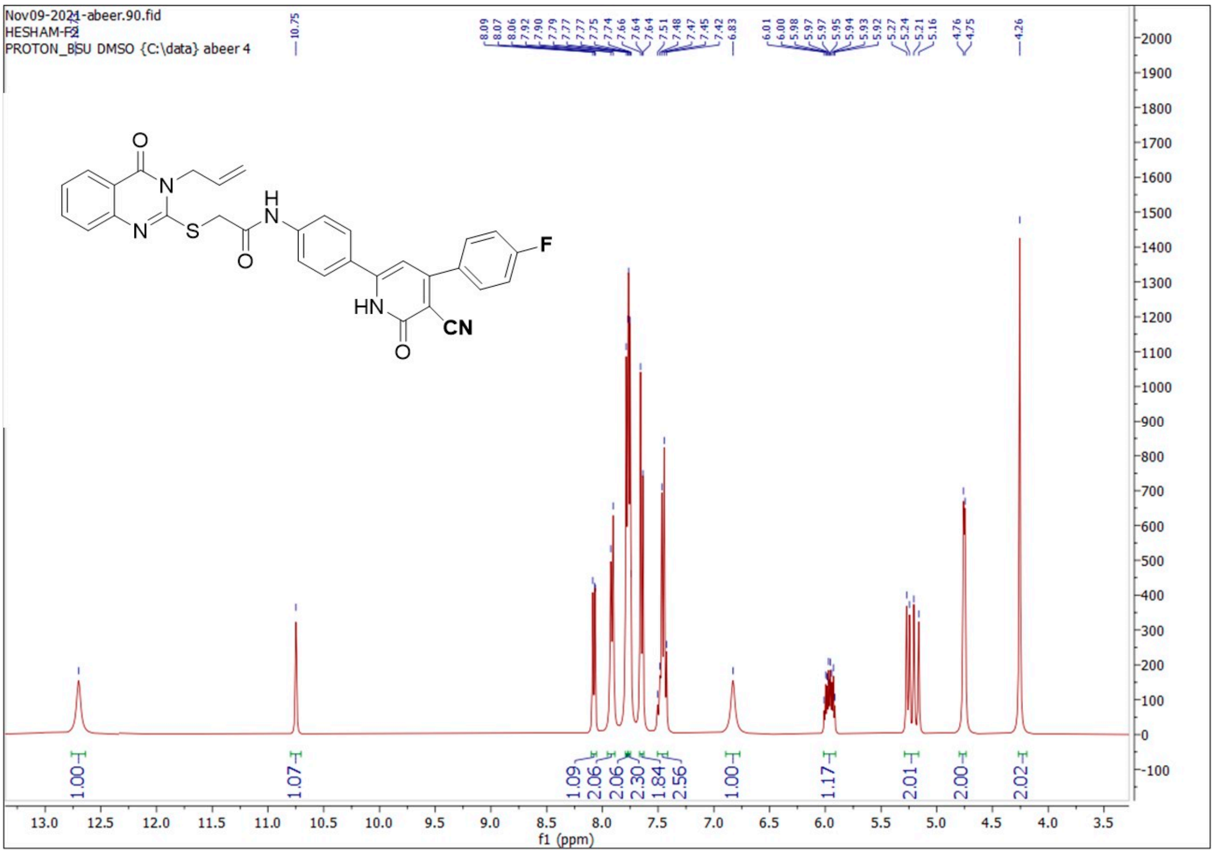
Nov09-2021-abeer.90.fid
HESHAM-F2
PROTON_BSU DMSO {C:\data} abeer 4
10.75
8.09 8.07 8.06 7.92 7.90 7.79 7.77 7.77 7.75 7.74 7.66 7.64 7.64 7.51 7.48 7.47 7.45 7.42 6.83
6.01 6.00 5.98 5.97 5.97 5.95 5.94 5.93 5.92 5.27 5.24 5.21 5.16 4.76 4.75 4.26
1.00 1.07 1.09 2.06 2.06 2.30 1.84 2.56 1.00 1.17 2.01 2.00 2.02
f1 (ppm)
HN
CN
F

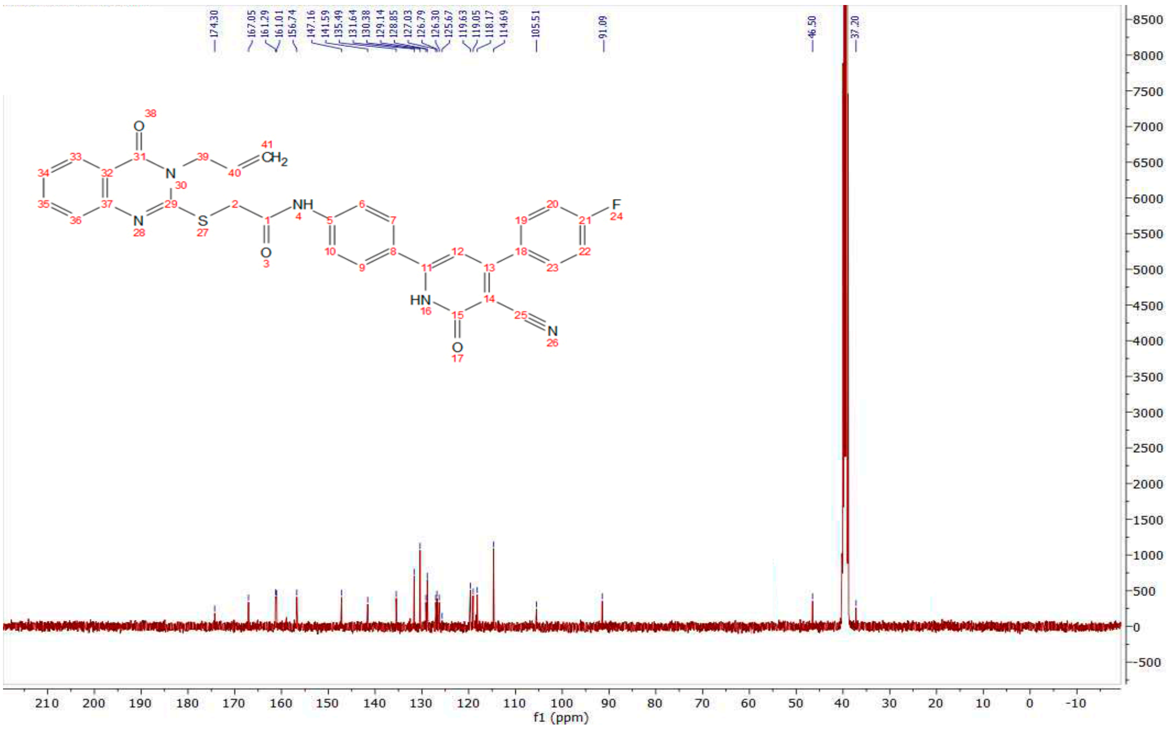
174.30 167.05 161.29 161.01 156.74 147.16 141.59 135.49 131.64 130.38 129.14 128.85 127.03 126.79 126.30 125.67 119.63 119.05 118.17 114.69 105.51 91.09 46.50 37.20
f1 (ppm)
NH
HN
F

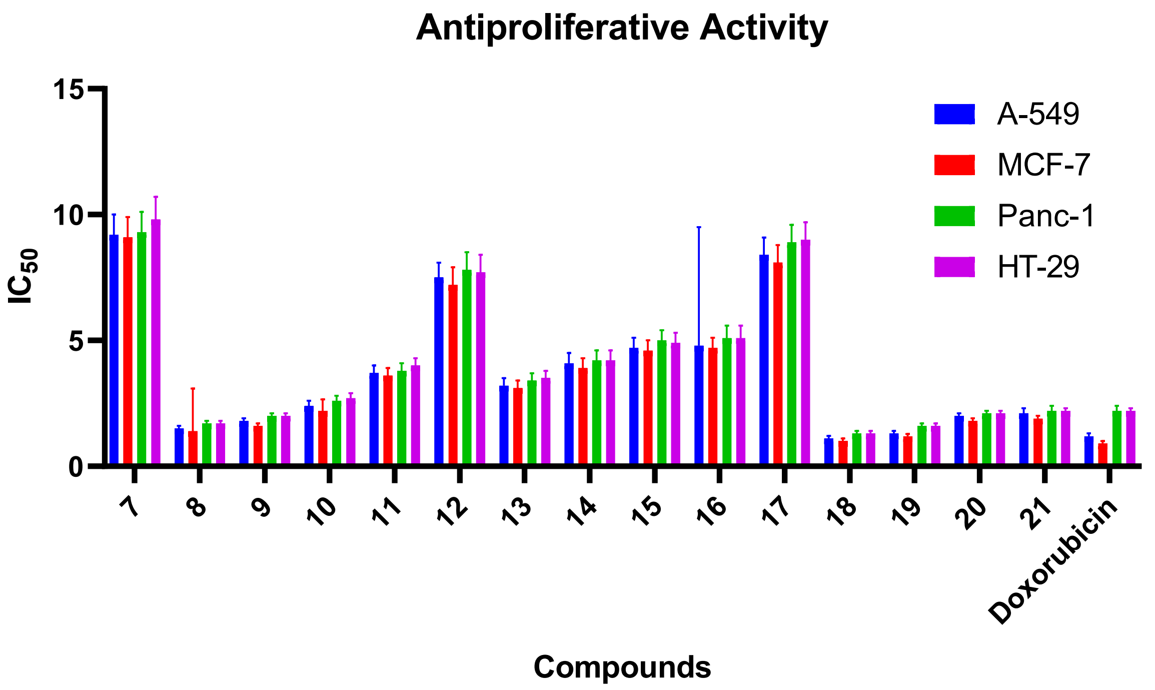

Antiproliferative Activity
IC50
15
10
5
0
A-549
MCF-7
Panc-1
HT-29
7
8
9
10
11
12
13
14
15
16
17
18
19
20
21
Doxorubicin
Compounds

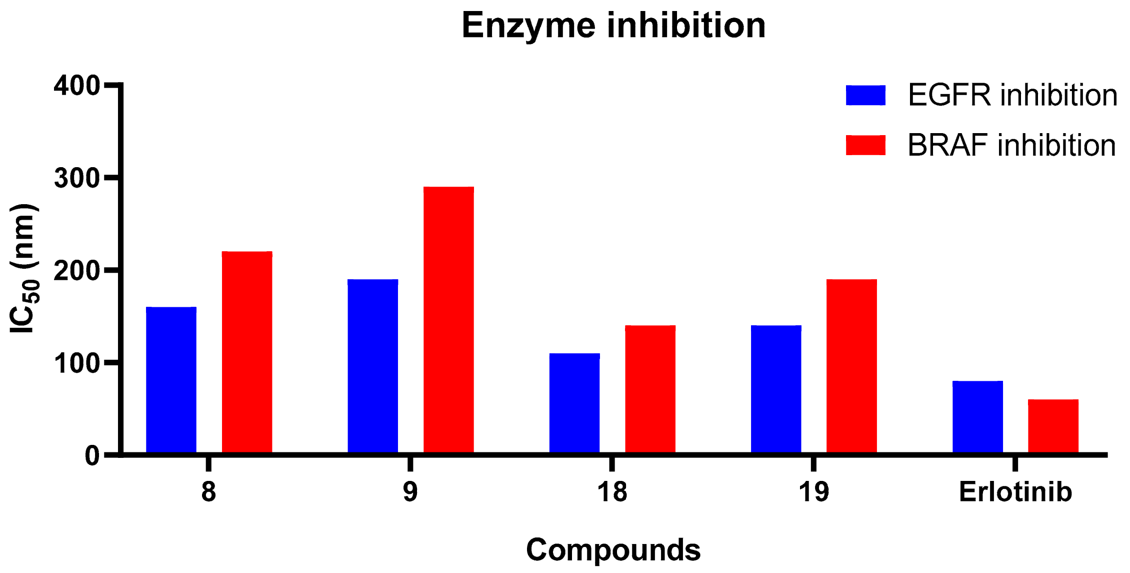

Enzyme inhibition
IC50 (nm)
400
300
200
100
0
EGFR inhibition
BRAF inhibition
8
9
18
19
Erlotinib
Compounds

Section A

3. Materials and Methods

3.1. Chemistry

General Details

Chemicals and solvents procured from Alfa Aesar, Sigma Aldrich, and El-Nasr Pharmaceutical Chemical Companies were utilized to synthesize the target compounds. The advancement of the reaction was observed by employing pre-coated TLC plates (Kieselgel 60f254 Merck), and the spots were identified by exposing them to UV light. Uncorrected melting points were measured with Stuart's electrothermal melting point apparatus. A JEOL JNM-GX-600 spectrometer (500MHz) located at the Faculty of Pharmaceutical Sciences, Umm Al-Qura University in Mecca, Saudi Arabia, and Faculty of Science, Zagazig University in Egypt, was used to perform 1HNMR spectra. The chemical shift (δ) in ppm relative to TMS (δ: 0 ppm) was taken as the internal standard, and DMSO-d6 was utilized as the solvent (δ: 2.5 ppm). The signal and coupling constant(*J*) in *Hz* were denoted by the following: s for singlet, d for doublet, t for triplet, q for quartet, and m for multiplet. Additionally, ^{13}C-NMR spectra were recorded on JEOL JNM-GX-150 in Switzerland (125 MHz), with chemical shift (δ) in ppm relative to TMS (δ: 0 ppm) as the internal standard and DMSO-$_{d6}$ as the solvent (δ: 39.50 ppm). Shimadzu GC/MS-QP5050A at the Regional Centre for Mycology and Biotechnology, Al-Azhar University, was used to conduct elemental analyses, and the results were found to be within ± 0.4% of the theoretical values4.2.

Biological evaluation

3.2.1. Cytotoxic activity using MTT Assay and evaluation of IC_{50}

3.2.1.1. MTT assay

MTT assay was performed to investigate the effect of the synthesized compounds on mammary epithelial cells (MCF-10A). The cells were propagated in medium consisting of Ham's F-12medium/ Dulbecco's modified Eagle's medium (DMEM) (1:1) supplemented with 10% foetal calfserum, 2 mM glutamine, insulin (10 µg/mL), hydrocortisone (500 ng/mL) and epidermal growth factor (20 ng/mL). Trypsin ethylenediamine tetra acetic acid (EDTA) was used to passage the cells after every 2-3 days. 96-well flat-bottomed cell culture plates were used to seed the cells at a density of 104 cells mL-1. The medium was aspirated from all the wells of culture plates after

24h followed by the addition of synthesized compounds (in 200 µL medium to yield a final concentration of 0.1% (v/v) dimethyl sulfoxide) into individual wells of the plates. Four wells were designated to a single compound. The plates were allowed to incubate at 37°C for 96 h. After wards, the medium was aspirated and 3-[4,5-dimethylthiazol-2-yl]-2,5-diphenyltetrazolium bromide (MTT) (0.4 mg/mL) in medium was added to each well and subsequently incubated for 3 h. The medium was aspirated and 150 µL dimethyl sulfoxide (DMSO) was added to each well. The plates were vortexed followed by the measurement of absorbance at 540 nm on a microplate reader. The results were presented as inhibition (%) of proliferation in contrast to controls comprising 0.1%DMSO.

3.2.1.2. Assay for antiproliferative effect

To explore the antiproliferative potential of compounds propidium iodide fluorescence assay was performed using different cell lines such as Panc-1 (pancreas cancer cell line), MCF-7 (breast cancer cell line), HT-29 (colon cancer cell line) and A-549 (epithelial cancer cell line), respectively. To calculate the total nuclear DNA, a fluorescent dye (propidium iodide, PI) is used which can attach to the DNA, thus offering a quick and precise technique. PI cannot pass through the cell membrane and its signal intensity can be considered as directly proportional to quantity of cellular DNA. Cells whose cell membranes are damaged or have changed permeability are counted as dead ones. The assay was performed by seeding the cells of different cell lines at a density of3000-7500 cells/well (in200µl medium) in culture plates followed by incubation for 24h at 37 °C in humidified 5% CO_2 / 95% air atmospheric conditions. The medium was removed; the compounds were added to the plates at 10 µM concentrations (in 0.1% DMSO) in triplicates, followed by incubation for 48h. DMSO (0.1%) was used as control. After incubation, medium was removed followed by the addition of PI (25 µl, 50µg/mL in water/medium) to each well of the plates. At -80 °C, the plates were allowed to freeze for 24 h, followed by thawing at 25oC. A fluorometer (Polar-Star BMG Tech) was used to record the readings at excitation and emission wavelengths of 530 and 620 nm for each well. The percentage cytotoxicity of compounds was calculated using the following formula: Where ATC= Absorbance of treated cells and AC= Absorbance of control. Erlotinib was used as positive control in the assay.

3.2.2. EGFR inhibitory assay

Baculoviral expression vectors including pBlueBacHis2B and pFASTBacHTc were used separately to clone 1.6 kb cDNA coding for EGFR cytoplasmic domain (EGFR-CD, amino acids645–1186). 5′ upstream to the EGFR sequence comprised a sequence that encoded (His)6. Sf-9cells were infected for 72h for protein expression. The pellets of Sf-9 cells were solubilized in a buffer containing sodium vanadate (100 µM), aprotinin (10 µg/mL), triton (1%), HEPES buffer(50mM), ammonium molybdate (10 µM), benzamidine HCl (16 µg/mL), NaCl (10mM), leupeptin (10 µg/mL) and pepstatin (10 µg/mL) at 0°C for 20 min at pH 7.4, followed by centrifugation for 20 min. To eliminate the nonspecifically bound material, a Ni-NTA super flow packed column was used to pass through and wash the crude extract supernatant first with 10mMand then with 100 mM imidazole. Histidine-linked proteins were first eluted with 250 and then with 500 mM imidazole subsequent to dialysis against NaCl (50 mM), HEPES (20 mM), glycerol (10%) and 1 µg/mL each of aprotinin, leupeptin and pepstatin for 120 min. The purification was performed either at 4 °C or on ice. To record autophosphorylation level, EGFR kinase assay was carried out based on DELFIA/Time-Resolved Fluorometry. The compounds were first dissolved in DMSO absolute, subsequent to dilution to appropriate concentration using HEPES(25 mM) at pH 7.4. Each compound (10 µL) was incubated with recombinant enzyme (10 µL, 5ng for EGFR, 1:80 dilution in 100 mM HEPES) for 10 min at 25oC, subsequent to the addition of5X buffer (10 µL, containing 2 mM MnCl2, 100 µM Na3VO4, 20 mM HEPES and 1 mM DTT)and ATP-MgCl2 (20 µL, containing 0.1 mM ATP and 50 mM MgCl2) and incubation for 1h. The negative and positive controls were included in each plate by the incubation of enzyme either with or without ATP-MgCl2. The liquid was removed after incubation and the plates were washed thrice using a wash buffer. Europium-tagged anti phosphotyrosine antibody (75 µL, 400 ng) was added to each well followed by incubation of 1h and then washing of the plates using buffer. The enhancement solution was added to each well and the signal was recorded at excitation and emission wavelengths of 340 at 615 nm. The autophosphorylation percentage inhibition by compounds was calculated using the following equation: Using the curves of percentage inhibition of eight concentrations of each compound, IC_{50} was calculated. The majority of signals detected by anti phosphotyrosine antibody were from EGFR because the enzyme preparation contained low impurities.4.2.3. BRAFV600E inhibitory assayV600E mutant BRAF kinase assay was performed to investigate the activity of tested compounds against BRAF. Mouse full-length GST-tagged

BRAFV600E (7.5 ng, Invitrogen, PV3849) was preincubated with drug (1 µL) and assay dilution buffer (4 µL) for 60 min at 25oC. In assay dilution buffer, a solution (5 µL) containing MgCl2 (30 mM), ATP (200 µM), recombinant human full length (200 ng) and N-terminal His-tagged MEK1 (Invitrogen) was added to start the assay, subsequent to incubation for 25 min at 25oC. The assay was stopped using 5X protein denaturing buffer (LDS) solution (5 µL). To further denature the protein, heat (70° C) was applied for 5 min.4-12% precast NuPage gel plates (Invitrogen) were used to carry out electrophoresis (at 200 V).10 µL of each reaction was loaded into the precast plates and electrophoresis was allowed to proceed. After completion of electrophoresis, the front part of the precast gel plate (holding hot ATP) was cut and afterwards cast-off. Dried gel was developed using a phosphor screen. A reaction without active enzyme was used as negative control while that containing no inhibitor served as positive control. To study the effect of compounds on cell-based pERK1/2 activity in cancer cells, commercially available ELISA kits (Invitrogen) were used according to manufacturer's instructions.

3.3. In Silico Studies

3.3.1. Docking Study

For the molecular docking study, we used BIOVIA Discovery Studio 2021 software v21.1.0.20.298. Protein Data Bank was used to obtain the crystal structures of EGFR (PDB ID: 1M17), BRAF kinase (PDB ID: 3OG7) underwent preparation for docking analysis through the Protein Preparation Wizard. Subsequently, ligands were mapped onto a three-dimensional model and subjected to energy minimization using LigPrep. To potentially improve binding, a receptor grid was created for the selected binding site using the Receptor Grid Generation Tool. Finally, the Glide tool was employed to evaluate both docking scores and various binding modes for the ligands.

3.3.2. In silico ADMET Analysis

ADMET studies were performed using BIOVIA I Discovery Studio 2016. The chemical structures of all compounds were imported, and ADMET descriptors were predicted using integrated models, including Lipinski's Rule of Five and assessments of absorption, distribution, metabolism,

excretion, and toxicity. The obtained results were analyzed to ascertain the drug-likeness and safety profiles of the compounds.

3.4. Statistical analysis

Computerized Prism 8 program was used to statistically analyzed data using one-way ANOVA test followed by Tukey's as post ANOVA for multiple comparison at $P \leq .05$. Data were presented as mean ± SEM.
